# *Smad4* Deficiency Promotes Pancreatic Cancer Immunogenicity by Activating the Cancer‐Autonomous DNA‐Sensing Signaling Axis

**DOI:** 10.1002/advs.202103029

**Published:** 2022-01-22

**Authors:** Wenjing Xiong, Wenzhuo He, Tiantian Wang, Shuai He, Feifei Xu, Zining Wang, Xiaojuan Wang, Hui Guo, Jianhua Ling, Huanling Zhang, Yongxiang Liu, Kaili Xing, Mengyun Li, Hongxia Zhang, Jiahui Li, Ningning Niu, Jing Xue, Qiuyao Zhan, Ze‐Xian Liu, Jin‐Xin Bei, Peng Huang, Jinyun Liu, Liangping Xia, Xiaojun Xia

**Affiliations:** ^1^ State Key Laboratory of Oncology in South China Collaborative Innovation Center for Cancer Medicine Sun Yat‐sen University Cancer Center Guangzhou 510060 China; ^2^ VIP Region Sun Yat‐sen University Cancer Center Guangzhou 510060 P. R. China; ^3^ Department of Molecular and Cellular Oncology The University of Texas MD Anderson Cancer Center Houston TX 77030 USA; ^4^ Department of Pancreatobiliary Surgery Sun Yat‐sen University Cancer Center Guangzhou 510060 P. R. China; ^5^ College of Food Science and Engineering Dalian Polytechnic University Liaoning 116034 P. R. China; ^6^ State Key Laboratory of Oncogenes and Related Genes Stem Cell Research Center Ren Ji Hospital Shanghai Jiao Tong University School of Medicine Shanghai 200127 P. R. China

**Keywords:** antitumor immunity, IFN‐I signaling, pancreatic cancer, SMAD4, STING

## Abstract

*Smad4*, a key mediator of the transforming growth factor‐*β* signaling, is mutated or deleted in 20% of pancreatic ductal adenocarcinoma (PDAC) cancers and significantly affects cancer development. However, the effect of *Smad4* loss on the immunogenicity and tumor immune microenvironment of PDAC is still unclear. Here, a surprising function of *Smad4* in suppressing mouse PDAC tumor immunogenicity is identified. Although *Smad4* deletion in tumor cells enhances proliferation in vitro, the in vivo growth of *Smad4*‐deficient PDAC tumor is significantly inhibited on immunocompetent C57BL/6 (B6) mice, but not on immunodeficient mice or CD8^+^ cell‐depleted B6 mice. Mechanistically, *Smad4* deficiency significantly increases tumor cell immunogenicity by promoting spontaneous DNA damage and stimulating STING‐mediated type I interferon signaling,which contributes to the activation of type 1 conventional dendritic cells (cDC1) and subsequent CD8^+^ T cells for tumor control. Furthermore, retarded tumor growth of *Smad4‐deficient* PDAC cells on B6 mice is largely reversed when *Sting* is codeleted, or when the cells are implanted into interferon‐alpha receptor‐deficientmice or cDC1‐deficientmice. Accordingly, *Smad4* deficiency promotes PDAC immunogenicity by inducing tumor‐intrinsic DNA damage‐elicited type I interferon signaling.

## Graphical abstract

The author's findings establish that the commonly inactivated tumor suppressor *Smad4* is a critical regulator of pancreatic ductal adenocarcinoma tumor immunogenicity by activating cancer‐autonomous DNA sensing signaling, and suggest a potential role of *Smad4* expression as a biomarker in developing future effective immunotherapy for PDACs.

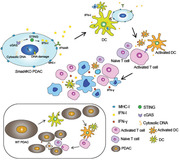

## 1 Introduction

Pancreatic ductal adenocarcinoma (PDAC) is the most common primary malignancy of the pancreas, and the 5‐year survival rate is extremely low at 3–5%. By 2030, PDAC is expected to become the second leading cause of cancer deaths.^[^ ^1^ ^]^ Early‐stage pancreatic cancer is usually asymptomatic and is not diagnosed until it has become locally advanced or metastatic. Despite a multitude of clinical treatments such as chemotherapy, immunotherapy and others, the prognosis of PDAC patients remains dismal, with a median overall survival of ≈4–6 months and has not been remarkably improved over the last 40 years.^[^ ^2^ ^]^ PDAC development and poor prognosis are partly attributed to complex interactions between tumor cells and stromal signals, immune responses. Since many genes mutation or deletion affect tumor cells’ growth and metastasis through autonomous or nonautonomous fashion, there is, therefore, a pressing need to further understand the impact of gene mutation on the immune microenvironment of PDAC.

Whole‐genome sequencing analysis revealed that *SMAD4*, *KRAS*, *TP53*, and *CDKN2a*/*INK4a* were among the most common mutated genes important for PDAC progression.^[^ ^3^ ^]^ Specifically, *SMAD4* (*DPC4*), encoding a common intracellular mediator of the transforming growth factor‐*β* (TGF‐*β*) and bone morphogenetic proteins (BMPs) superfamily,^[^ ^4^ ^]^ is mutated or deleted in over 20% of pancreatic cancer patients.^[^ ^5^ ^]^ It is well established that *Smad4* plays a critical role in TGF‐*β* signaling pathway activation to regulate a variety of critical processes, including fibrosis orchestrates,^[^ ^6^ ^]^ tumor development ^[^ ^7^ ^]^ and immunotherapy,^[^ ^8^ ^]^ immune function,^[^ ^9^ ^]^ and wound healing.^[^ ^10^ ^]^ *Smad4* is conventionally considered as a tumor suppressor gene by blocking tumor cells’ mitogenic signals.^[^ ^7^ ^]^ On the other hand, previous findings have convincingly established the TGF‐*β*/SMAD signaling pathway as a major inhibitory signal for immune response, such as inhibiting the function of cytotoxic T cells ^[^ ^11^ ^]^ and natural killer (NK) cells,^[^ ^12^ ^]^ inducing FOXP3^+^ regulatory T cells recruitment,^[^ ^13^ ^]^ and converting antigen‐presenting cell function from immune activation to tolerance.^[^ ^14^ ^]^ Interestingly, mouse model studies identified genomic instability and proinflammatory phenotype in skin‐ and head‐and‐neck‐specific *Smad4* knockout mice.^[^ ^15^ ^]^ Consistently, recent studies observed an inflamed tumor microenvironment in SMAD4‐deficient PDAC mouse tumor models.^[^ ^16^ ^]^ Nevertheless, the role of *Smad4* in regulating antitumor immune response for PDAC is not well understood.

The tumor microenvironment includes cancer cells and many types of stromal cells, including immune cells, which play critical role in antitumor immunity. Tumor cells or stroma cells release cytokine or chemokine to recruit immune cells (such as NK, dendritic cells (DC) and T cells) for tumor control. Especially, early reports have demonstrated that type I interferon (IFN‐I) promotes antitumor immunity.^[^ ^17^ ^]^ IFN‐Is are cytokines that play a pivotal role in limiting pathogenic infection and tumor growth.^[^ ^18^ ^]^ For anticancer therapy, both cancer cell‐autonomous and immune cell‐derived IFN‐I have been related to breast cancer patient response to chemotherapy or mouse melanoma response to anti‐ programmed cell death protein 1(PD1) therapy.^[^ ^19^ ^]^ Furthermore, IFN‐I is critical for the efficacy of radiation therapy and immunotherapies such as PD‐1 blockade.^[^ ^20^ ^]^ Pathogen‐derived nucleic acid (such as viral DNA/RNA) or self‐DNA from genomic DNA damage is sensed by intracellular nucleic acid sensors—cyclic GMP‐AMP synthase (namely cGAS). Then, cGAS binds to DNA fragment and synthesizes 2'3'‐Cyclic GMP‐AMP (cGAMP), which then activates STING (also known as *Mita*, *Eris* and *Mpys*) to interact with the downstream TANK‐binding kinase 1 (TBK1) and interferon regulator factor 3 (IRF3), and finally activates IFN‐I pathway gene expression.^[^ ^21^ ^]^ Type I IFNs then activate the Janus kinase (JAK)‐signal transducer and activator of transcription (STAT) pathway and induce an IFN‐stimulated gene (ISG) transcriptional program ^[^ ^22^ ^]^ which can regulate multiple physiological functions.^[^ ^23^ ^]^

Here, we showed that *Smad4* deletion in mouse PDAC tumors resulted in enhanced tumor immunogenicity. Loss of *Smad4* in PDAC tumor cells inhibited tumor growth in vivo in a manner dependent on CD8^+^ T cells and type I conventional dendritic cells (cDC1). Increased level of spontaneous DNA damage in SMAD4‐deficient PDAC cells stimulated STING‐mediated IFN‐I signaling to promote tumor cell major histocompatibility complex‐I (MHC‐I) expression and cDC1‐mediated antigen cross‐presentation, which together boosted antitumor immunity.

## 2 Results

### 2.1 Loss of *Smad4* in Mouse PDAC Promoted Tumor Cell Growth In Vitro but Inhibited Tumor Growth In Vivo

**Figure 1:**
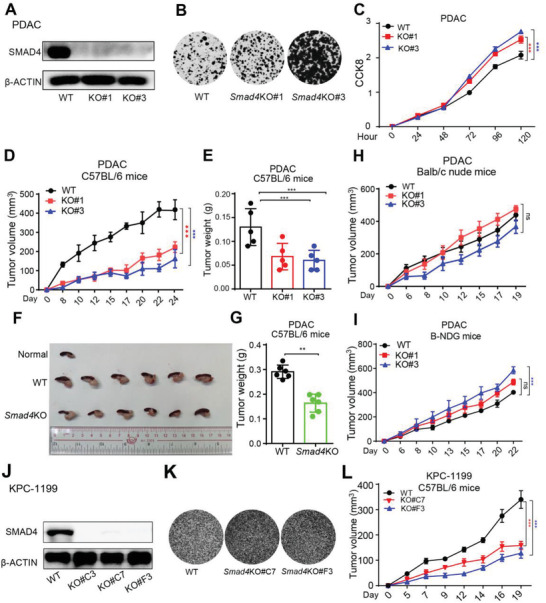
*Smad4* deletion promoted PDAC tumor cells growth in vitro but inhibited tumor growth on immune‐competent mice. A) Western blot analysis showing SMAD4 protein expression in Wild‐type (WT) and two independent clones of *Smad4*KO PDAC cells (KO#1, KO#3). B) PDAC cells were subjected to a 2D‐colony‐formation assay, and the colonies were stained with 2‐(4‐iodo‐phenyl)‐3‐(4‐nitrophenyl)‐5‐phenyl‐2H‐tetrazolium chloride (INT). C) Cell Counting Kit‐8 (CCK8) assay measuring the proliferation of PDAC cells at indicated time points. Data are presented as means ± SEM; three independent experiments were performed. ***, *p* < 0.001; by two‐way ANOVA test. D) Equal numbers of WT or *Smad4*KO PDAC cells were subcutaneously inoculated into B6 mice, and the tumor growth was monitored at the indicated time points. Data are presented as mean ± SEM; *n* = 5 for each group.***, *p* < 0.001; by two‐way ANOVA test. E) B6 mice were inoculated with WT or *Smad4*KO PDAC cells, and primary tumor weight was determined 22 days after inoculation. Results are presented as mean ± SEM; *n* = 5 for each group; ***, *p* < 0.001; by Mann–Whitney test. F,G) Tumor cells were implanted orthotopically into pancreas of B6 mice. The mice were euthanized 9 days after inoculation and the tumor tissues were harvested and photographed (F). Tumor weight was shown in (G). Data are presented as mean ± SEM, *n* = 6 for each group, **, *p* < 0.01; by Mann–Whitney test. H,I) Equal numbers of WT or *Smad4*KO PDAC cells were subcutaneously inoculated into nude mice (H) and B‐NDG mice ( I), and tumor growth was monitored at the indicated time points. Data are presented as mean ± SEM; *n* = 5 for each group. ns, not significant; ***, *p* < 0.001; by two‐way ANOVA test. J–L) Western blot, 2D‐colony‐formation assay and subcutaneous tumor experiments were performed using WT or *Smad4*KO KPC‐1199 cell line. Data are presented as mean ± SEM; for animal experiment, *n* = 5 for each group. ***, *p* < 0.001; by two‐way ANOVA test.

To elucidate the role of SMAD4 in antitumor immunity, we used CRISPR/Cas9 technology to knockout *Dpc4* gene encoding SMAD4 in PDAC, a murine pancreatic ductal adenocarcinoma cell line derived from pancreatic tumor tissue of an *LSL‐Kras^G12D/^* ^+^, *LSL‐Trp53^R172H/^* ^+^, *Pdx‐1‐Cre* (KPC) mouse (**Figure** **1**A), and two individual clones were used for this study. The *Smad4* knockout (*Smad4*KO) PDAC cells grew significantly faster in vitro than WT cells (Figure 1B,C). Next, to elucidate the role of *Smad4* in tumor growth in vivo, we subcutaneously inoculated equal numbers of WT or *Smad4*KO cells into syngeneic C57BL/6 (B6) mice and monitored the tumor growth. Unexpectedly, *Smad4* deficiency significantly impaired tumor growth in immune‐competent B6 mice (Figure 1D,E). Consistently, *Smad4*KO tumors also grew slower than WT tumors in the orthotopic implantation model (Figure 1F,G). On the contrary, the tumor growth of *Smad4*KO cells was comparable or slightly faster than that of WT when inoculated into T cell‐deficient nude mice (Figure 1H) or severely immunodeficient B‐NDG (NOD‐*Prkdc^scid^ IL2rg^tm1^*/Bcgen) mice (Figure 1I). Furthermore, another murine pancreatic cancer cells line, KPC‐1199, derived from pancreatic tumor tissue of an KPCmouse (also known as FC 1199), underwent *Smad4*‐knockout and two individual clones were produced. Similar to *Smad4*KO PDAC cells, KPC‐1199‐*Smad4*KO cells‐derived tumors grew slower than wild‐type tumors in the immune‐competent B6 mice (Figure 1J–L).

Thus, our results indicated that loss of *Smad4* in murine pancreatic cancer cells promoted tumor cell growth in vitro but paradoxically inhibited tumor growth in vivo, likely by engaging T cell‐mediated antitumor immunity.

### 2.2 Slow Growth of *Smad4*KO PDAC Tumor Was Dependent on CD8^+^ T Cell‐Mediated Antitumor Immunity

**Figure 2:**
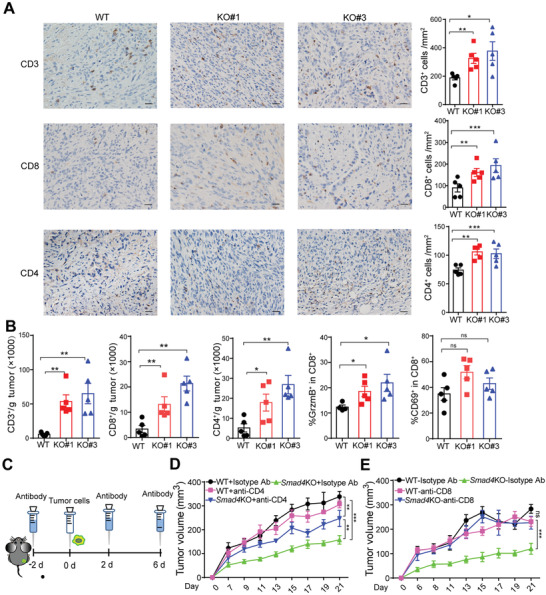
Loss of *Smad4* in PDAC cells induced antitumor immune response in host mice. A) Left, representative IHC staining images of the WT and *Smad4*KO PDAC tumor tissues. Right, number of CD3^+^, CD8^+^ and CD4^+^ cells per mm^2^; *n* = 5, scale bar = 50 µm. *, *p* < 0.05; **, *p* < 0.01; ***, *p* < 0.001; by Mann–Whitney test. B) Single‐cell suspensions isolated from WT or *Smad4*KO PDAC tumors were stained and gated with CD45^+^ CD3^+^ cells, CD45^+^ CD8^+^ cells, and CD45^+^ CD4^+^ cells, and the cell numbers per gram of tumors were counted. Data are presented as mean ± SEM, *n* = 5, ns, not significant, *, *p* < 0.05; **, *p*<0.01; by Mann–Whitney test. C) The schedule for deletion antibody administration in mouse PDAC tumor model. D,E) B6 mice were pretreated with isotype control antibody, anti‐CD4 or anti‐CD8 depletion antibody, then the tumor growth of WT or *Smad4*KO PDAC was monitored at the indicated time points. Data are presented as mean ± SEM; *n* = 5 mice for each group. ns, not significant; **, *p* < 0.01; ***, *p* < 0.001; by two‐way ANOVA test.

As *Smad4*KO PDAC tumors grow differently on immune‐competent mice versus immunodeficient mice, we hypothesized that host immune response might contribute to the growth difference in vivo. We then collected PDAC and KPC‐1199‐derived tumor tissues to examine T cell infiltration by immunohistochemical (IHC) staining and flow cytometry (FCM) analysis. Massive infiltration of CD3^+^, CD4^+^, and CD8^+^ cells was observed in *Smad4*KO tumor tissues by IHC staining (**Figure** **2**A; Figure S1C, Supporting Information). In line with the IHC staining result, FCM analysis using single‐cell suspension from tumor tissues (Figure S1A, Supporting Information) also showed increased levels of T cell infiltration and CD8^+^ T cell activation in the *Smad4*KO group compared with WT tumors (Figure 2B; Figure S1D, Supporting Information). As the TGF‐*β*/SMAD4 signaling has been reported to influence regulatory T (Treg) ^[^ ^13^ ^]^ and T helper 17 (Th17) ^[^ ^24^ ^]^ cell differentiation, we checked the percentage of Treg (gated as CD4^+^FOXP3^+^ population) and Th17 cells (gated as CD4^+^ROR*γ*T^+^ population) in tumor‐infiltrating CD4^+^ cells, and found no difference between WT and KO group (Figure S1B,D, Supporting Information).

To verify if tumor‐infiltrating T cells were responsible for *Smad4* loss‐induced tumor suppression, we depleted T cells via intraperitoneal injection of anti‐CD8 or anti‐CD4 neutralizing antibodies prior to tumor cell inoculation (Figure 2C). Depletion of T cells was confirmed by FCM analysis (Figure S2A, Supporting Information). Strikingly, *Smad4KO*‐mediated tumor suppression was totally abolished after CD8^+^ cells were depleted and partially attenuated when CD4^+^ cells were depleted (Figure 2D,E). Although an increased number of infiltrating NK cells (sorted as CD3^−^ NK1.1^+^ population) was also identified in *Smad4*KO tumors (Figure S2B, Supporting Information), depletion of NK cells only accelerated *Smad4*KO cells growth at an early stage, and the growth of *Smad4*KO tumors became slower after 11 days (Figure S2C,D, Supporting Information). These results suggest that, T cells, especially CD8^+^ T cells, but not NK cells, may play a pivotal role in *Smad4*KO‐induced tumor immune response. Together, these data suggest that in vivo tumor inhibition by *Smad4* deletion requires T cell‐mediated adaptive immune responses.

### 2.3 *Smad4* Deficiency Enhanced the Immunogenicity of PDAC Tumor Cells

**Figure 3:**
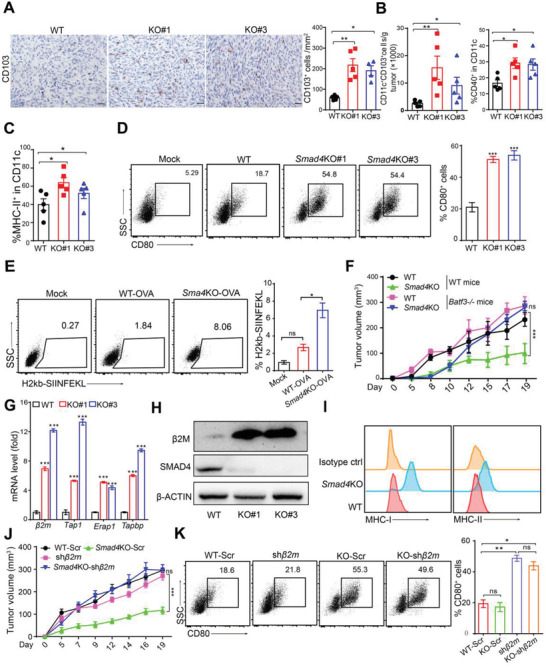
Deletion of *Smad4* in PDAC tumor cells enhanced tumor immunogenicity. A) Left, representative IHC staining images of the WT and *Smad4*KO PDAC tumor tissues. Right, bar graph showing the count of CD103^+^ cells. Scale bars = 50 µm. Data are presented as mean ± SEM. *, *p* < 0.05; **, *p* < 0.01; by Mann–Whitney test. B,C) DC infiltration and activation levels in WT or *Smad4*KO PDAC tumors identified by FCM analysis. Data are presented as mean ± SEM; *, *p* < 0.05; **, *p* < 0.01; by Mann–Whitney test. D) DCs cocultured with tumor cells (WT or *Smad4*KO) for 24 h, and the percentage of CD80^+^ DCs was counted by FCM analysis. Three independent experiments were performed. Data are presented as mean ± SEM. ***, *p* < 0.001; by Mann–Whitney test. E) DCs were cocultured with PDAC‐OVA (WT or *Smad4*KO) for 24 h, and then subjected to FCM analysis for surface expression of MHC‐I‐OVA (H2k^b^‐SIIFEKL) complex. Three independent experiments were performed. Data are presented as mean ± SEM. ns, not significant; *, *p* < 0.05; by Mann–Whitney test. F) WT or *Smad4*KO PDAC cells were inoculated on *Batf3* ^+/+^ (WT) mice or *Batf3* ^−/−^ mice, and then tumor growth was monitored at the indicated time points. Data are presented as mean ± SEM; *n* = 6 per group. ns, not significant; ***, *p* < 0.001; by two‐way ANOVA test. G) Real‐time qPCR analysis of expression levels of antigen presentation machinery molecules in WT or *Smad4*KO PDAC cells. Data are presented as mean ± SEM; three independent experiments were performed. ***, *p* < 0.001; by Mann–Whitney test. H) Western blot showing *β*2M protein levels in WT or *Smad4*KO PDAC cells. I) FCM analysis of the cell surface expression of MHC‐I and MHC‐II molecules in WT or *Smad4*KO PDAC cells. J) B6 mice were subcutaneously inoculated with WT or *Smad4*KO PDAC cells expressing scrambled (Scr) or *β2m*‐specific shRNA (sh*β2m*), and then tumor growth was recorded. Data are presented as mean ± SEM; *n* = 6 per group. ns, not significant; ***, *p* < 0.001; by two‐way ANOVA test. K) DCs were cocultured with PDAC cells for 24 h, and the percentage of CD80^+^ DCs was analyzed by FCM analysis. Data are presented as mean ± SEM. ns, not significant; *, *p* < 0.05; **, *p* < 0.01; by Mann–Whitney test.

Antitumor T cell priming requires antigen presentation from DCs which recognizes and presents the antigen of tumor cell. Recent findings suggest that in vivo cross‐presentation of tumor‐derived antigens is mainly dependent on cDC1s, which often show CD103 expression in tumor tissues.^[^ ^25^ ^]^ Indeed, the levels of tumor‐infiltrating CD11c^+^CD103^+^ cells and activated DCs (gated as CD40^+^CD11c^+^ and MHC‐II^+^CD11c^+^) were both increased in *Smad4*KO PDAC (**Figure** **3**A–C; Figure S3A, Supporting Information) and KPC‐1199 tumors (Figure S3B,C, Supporting Information), suggesting enhanced intratumoral DC activation.

We next evaluated tumor‐induced DC activation in vitro. We cocultured PDAC tumor cells with FMS‐like tyrosine kinase 3 ligand (Flt3L‐induced DCs, consisting of a mixture of cDC1 and cDC2 cells,^[^ ^26^ ^]^ and then the expression of costimulatory molecules CD80 and CD86 on DCs were examined. Our results showed that DCs cocultured with *Smad4*KO tumor cells showed higher levels of CD80^+^ (Figure 3D) and CD86^+^ (Figure S3D, Supporting Information) cells than those cocultured with WT tumor cells. To further evaluate antigen cross‐presentation, we constructed PDAC‐ovalbumin (OVA) and PDAC‐*Smad4*KO‐OVA cells by stable transfection of model antigen OVA and performed FCM analysis to detect DCs and OT‐1 T cell activation after coculture with tumor cells. Consistently, the PDAC‐*Smad4*KO‐OVA cells‐priming induced a higher percentage of DCs expressing MHC‐I‐bound SIINFEKL (a CD8 epitope peptide from OVA) (Figure 3E) and T cells expressing IFN‐*γ* and Granzyme B (Figure S3E, Supporting Information) than that of DCs and T cells primed by PDAC‐WT‐OVA. Furthermore, B3Z cells, an OVA‐specific T cell hybridoma cell line ,^[^ ^27^ ^]^ produced higher levels of IL‐2 when cocultured with DCs primed with PDAC‐*Smad4*KO‐OVA cells than those primed with WT cells (Figure S3F, Supporting Information), suggesting enhanced antigen processing. Finally, when inoculated on Basic Leucine Zipper ATF‐Like Transcription Factor 3 (*Batf3*)^−/−^ mice lacking cDC1 cells, the tumor growth disadvantage of *Smad4*KO PDAC and KPC‐1199 tumors was nearly completely reversed (Figure 3F; Figure S3G, Supporting Information). Collectively, these results demonstrated that *Smad4*KO‐mediated tumor inhibition was caused by cDC1 cells‐mediated antigen cross‐presentation and subsequent CD8^+^ T cell‐mediated antitumor response.

Given that *Smad4* deletion in tumor cells promoted the sensitivity to host immune response, we speculated that *Smad4* deletion might enhance the tumor cells’ immunogenicity, which promoted immune recognition of tumor cells. Therefore, we evaluated the expression levels of antigen presentation machinery‐related molecules on tumor cells. The mRNA levels of mouse *beta‐*2 microglobulin (*β2m*), an essential component of MHC‐I, as well as the genes directing peptide cleavage (*Erap1*), peptide transporters (*Tap1*) and transporter‐MHC interactions (*Tapbp*) were all increased in *Smad4*KO cells (Figure 3G; Figure S3H, Supporting Information). Consistently, the protein levels of *β*2M, MHC‐I and MHC‐II in *Smad4*KO cells were drastically increased compared to that of WT cells (Figure 3H,I; Figure S4E, Supporting Information).

To confirm whether the elevated expression of antigen presentation machinery on tumor cells is essential for *Smad4*KO‐mediated tumor inhibition, we knocked down *β2m* expression by shRNA in *Smad4* KO PDAC cells (Figure S3I, Supporting Information). Consequently, MHC‐I (H2k^b^ for B6 mouse) level markedly decreased in *β2m*‐silenced cells (Figure S3J, Supporting Information). Strikingly, *Smad4*KO‐mediated tumor growth inhibition on B6 mice was reversed when *β2m* expression was silenced (Figure 3J), confirming the essential role of MHC‐I for T cell‐mediated tumor suppression. However, DC activation induced by *Smad4*KO PDAC cells cocultured in *vitro* was not affected by *β2m* knockdown (Figure 3K; Figure S3K, Supporting Information), suggesting that *Smad4*KO tumor cells induced DC activation through mechanisms other than increased MHC‐I expression. Together, these results indicated that *Smad4* deletion in PDAC cells enhanced immunogenicity through: 1, increasing antigen presentation machinery molecule expression on tumor cells; 2, promoting DC activation.

### 2.4 *Smad4* Deficiency Activated Type I IFN Signaling in PDAC Tumor Cells

We next explored how *Smad4* deficiency in tumor cells increased MHC‐I expression. A recent study reported that autophagy in PDAC cells could induce MHC‐I degradation to promote tumor immune evasion, and blockade of autophagy in PDAC tumor cells restored surface level of MHC‐I and improved antigen presentation, leading to enhanced antitumor immunity in syngeneic host mice.^[^ ^28^ ^]^ Here, we examined the microtubule‐associated protein light chain 3beta (LC3B) protein level as a marker for autophagy level, ^[^ ^29^ ^]^ and found that *Smad4*KO cells had higher levels of LC3B expression than WT cells (Figure S4A, Supporting Information). However, *β*2M or MHC‐I expression level was not changed in WT PDAC cells when treated with either autophagy inducer (Rapamycin) or autolysosome inhibitor (Bafilomycin A1), and was modestly increased in *Smad4*KO cells by bafilomycin A1 treatment but not by Rapamycin treatment (Figure S4B,C, Supporting Information). These findings suggest that *Smad4*KO‐induced *β*2M or MHC‐I upregulation in PDACs was not likely due to autophagy inhibition.

**Figure 4:**
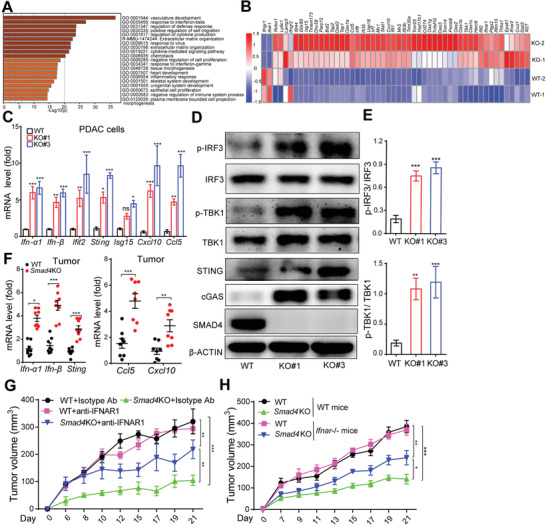
Loss of *Smad4* activated type I IFN signaling pathway in PDAC tumor cells. A) Pathway enrichment analysis on RNA‐seq results from WT and *Smad4*KO PDACs. B) Heat maps of gene expression levels in WT and *Smad4*KO PDAC cells. C) Real‐time PCR analysis of mRNA levels of ISGs in WT and *Smad4*KO PDAC cells. Data are presented as mean ± SEM; ns, not significant; *, *p* < 0.05; **, *p* < 0.01; ***, *p* < 0.001; by Mann–Whitney test. D,E) Western blot analysis of protein expression levels in WT and *Smad4*KO PDAC cells (D). Bar graph showing the quantified values of IRF3 or TBK1 phosphorylation levels from three independent experiments (E). Data are presented as means ± SEM of three independent experiments. **, *p* < 0.01; ***, *p* < 0.001; by Mann–Whitney test. F) RNA from WT and *Smad4*KO PDAC tumor tissues was isolated and real‐time PCR was performed to detect expression of ISGs, as indicated. Data are presented as mean ± SEM; *n* = 8 tumors in each group. *, *p* < 0.05; **, *p* < 0.01; ***, *p* < 0.001; by Mann–Whitney test. G) WT or *Smad4*KO PDAC tumor growth on B6 mice with IFNAR blockade by intraperitoneal injection of anti‐IFNAR antibody. Data are presented as mean ± SEM; *n* = 5 for each group. **, *p* < 0.01; ***, *p* < 0.001; by two‐way ANOVA test. H) WT or *Smad4*KO PDAC tumor growth on WT or *Ifnar* ^−/−^ mice. Data are presented as mean ± SEM; *n* = 5 for each group. *, *p* < 0.05; **, *p* < 0.01; ***, *p* < 0.001; by two‐way ANOVA test.

To comprehensively delineate the mechanism of *Smad4*KO‐mediated tumor suppression or immunogenicity, we performed RNA‐sequencing to analyze the global gene expression difference between WT cells and *Smad4*KO cells. Pathway enrichment analysis (https://metascape.org) identified that the "response to IFN‐*β*" signaling pathway was among the most upregulated pathways in *Smad4*KO cells (**Figure** **4**A). Next, we profiled the gene expression associated with the IFN‐I signaling pathway. We identified that IFN pathway‐related transcription factors (for example, *Stat1*, *Stat3*, *Sting*, *Irf7*, and *Irf9*) and ISGs (for example, *Ifit1*, *Ifit2*, *Cxcl1*, *Cxcl10*, *Ccl5*, *Bst2*, and *Mx1*), were all upregulated (Figure 4B). Since the expression level of *Ifnar* (type I IFN receptor), but not the *Ifngr* (type II IFN receptor) or *Ifnlr* (type III IFN receptor), was increased (Figure 4B), we reasoned that type I IFN signaling pathway might play a role in *Smad4*KO‐mediated tumor inhibition. Quantitative PCR confirmed the upregulation of IFN‐I and ISG genes expression in *Smad4*KO cells (Figure 4C). Interestingly, we also identified a key IFN‐I pathway regulator gene, *Tmem173* (encoding STING), among these upregulated genes. Consistently, *Smad4*KO PDAC and KPC‐1199 cells exhibited increased levels of IRF3 and TBK1 phosphorylation, with concurrent upregulation of cGAS and STING (Figure 4D,E; Figure S4D,E, Supporting Information). Importantly, PDAC tumors derived from *Smad4*KO cells exhibited significantly increased levels of IFN‐I and ISGs (Figure 4F; Figure S4F, Supporting Information).

Since IFN‐*α*/*β* signaling is known as a critical signal for T cell cross‐priming,^[^ ^30^ ^]^ we next investigated the role of IFN‐I signaling in *Smad4*KO‐induced tumor immunogenicity in vivo. Blocking IFN‐*α*/*β* receptor (IFNAR) by anti‐IFNAR neutralizing antibody significantly promoted *Smad4*KO but not WT tumor growth in B6 mice (Figure 4G; Figure S4G, Supporting Information). FCM assay also showed that T cells and DC cells infiltration and activation levels were dramatically decreased when IFNAR was blocked (Figure S4H,I, Supporting Information). Consistently, *Smad4*KO PDAC tumor growth was significantly faster on *Ifnar^−/−^* mice than on WT mice while the WT tumor growth was largely comparable between WT or *Ifnar^−/−^* mice (Figure 4H). Together, we figured that the IFN‐I signaling pathway played a critical role in *Smad4*KO‐mediated tumor inhibition.

A previous study reported an increased level of inflammation and TGF‐*β* expression in head and neck epithelia of tissue‐specific *Smad4*‐deficient mice.^[^ ^15a^ ^]^ Consistently, we also identified increased levels of TGF‐*β* mRNA in *Smad4*KO PDAC cells (Figure S5A, Supporting Information). However, the difference in expression levels of *Ifn‐β* and MHC‐associated molecules between WT and *Smad4*KO cells was largely unchanged upon TGF‐*β* treatment (Figure S5A, Supporting Information), suggesting that *Smad4*KO‐induced IFN‐I upregulation is not dependent on conventional TGF‐*β* signaling.

### 2.5 Tumor‐Intrinsic STING Is Required for *Smad4*KO‐Induced PDAC Immunogenicity

Early reports identified that *Smad4*‐knockdown human pancreatic cancer cells or mouse keratinocytes with *Smad4* gene knockout exhibited increased spontaneous DNA damage and reduced DNA repair.^[^ ^15^, ^31^ ^]^ Consistent with the genetic knockout mouse model, the protein levels of classical DNA damage markers, *γ*‐H2AX and RAD51, were markedly increased in *Smad4*KO cells (Figure S5B,C, Supporting Information). Moreover, the cytoplasmic DNA levels were also increased (Figure S5D, Supporting Information). Noteworthy, high‐level DNA damage may induce gene mutations that can potentially generate mutagenic peptides distinct from self and form so‐called neoantigen, which may contribute to the immunogenic phenotype.^[^ ^32^ ^]^ Whole exon sequencing revealed that *Smad4*KO cells showed slightly, but not significantly, higher levels of single nucleotide variants (SNVs) and tumor mutant burden (TMB) compared with WT cells on early passage (P3) or late passage (P55) (Figure S5E,F and Tables S1 and S2, Supporting Information). Thus, increased DNA damage did not cause a high mutation burden in *Smad4*KO cells. Instead, we speculated that damaged DNA fragments from nucleus or mitochondria may leak to the cytoplasm to stimulate intracellular DNA sensors such as cGAS/STING to promote IFN *α*/*β* activation. Therefore, DNA damage‐induced IFN‐I activation, but not mutation accumulation, may contribute to the increased immunogenicity of *Smad4*KO tumor cells.

**Figure 5:**
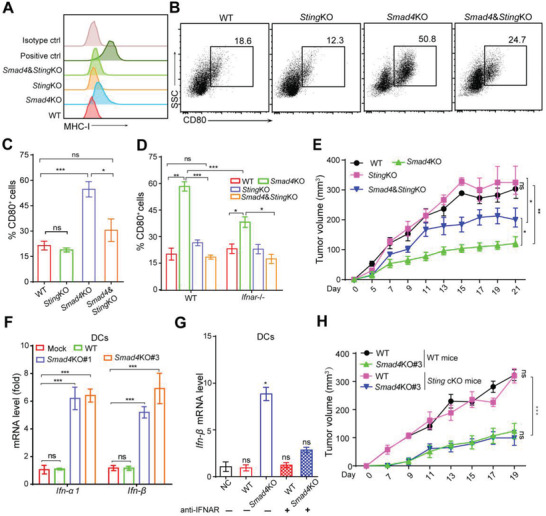
Tumor‐intrinsic STING signaling in *Smad4*KO cells mediated tumor suppression. A) FCM analysis of the surface expression of MHC‐I in PDAC cells. B,C) cDCs were cocultured with PDAC tumor cells (*Sting*KO or *Smad4*&*Sting*KO) for 24 h, after which CD80^+^ DCs was identified by FCM analysis (B) and the percentage was counted (C). Data are presented as mean ± SEM and three independent experiments were performed. ns, not significant, *, *p* < 0.05; ***, *p* < 0.001; by one‐way ANOVA test followed by Tukey's multiple comparison test. D) Tumor cells with indicated genotypes were cocultured with WT or *Ifnar* ^−/−^ cDCs for 24 h, and the percentage of CD80^+^ DCs is shown. Three independent experiments were performed. Data are presented as mean ± SEM. ns, not significant, *, *p* < 0.05; **, *p* < 0.01, ***, *p* < 0.001; by one‐way ANOVA test followed by Tukey's multiple comparison test. E) Tumor growth of PDAC cells with indicated genotypes on B6 mice. Data are presented as mean ± SEM; *n* = 5 for each group. *, *p* < 0.05; **, *p* < 0.01; by two‐way ANOVA test. F) Real‐time qPCR analysis of *Ifn‐α*/*β* expression of cDCs after coculture with PDAC tumor cells for 24h. Data are presented as mean ± SEM. Three independent experiments were performed. ns, not significant, ***, *p* < 0.001; by one‐way ANOVA test followed by Tukey's multiple comparison tests. G) Real‐time qPCR analysis of *Ifn‐β* expression of cDCs with or without anti‐IFNAR pretreatment before coculture with PDAC tumor cells. Data are presented as mean ± SEM. Three independent experiments were performed. ns, not significant, *, *p* < 0.05; by one‐way ANOVA test followed by Tukey's multiple comparison tests. H) Tumor growth of WT or *Smad4*KO PDAC cells on *Sting* ^+/+^ (WT) mice or DC‐specific *Sting* KO (*Sting* ^cKO^) mice. Data are presented as mean ± SEM; *n* = 5 for each group. ns, not significant, ***, *p* < 0.001; by two‐way ANOVA test.

Since STING is a major regulator for intracellular DNA‐induced IFN‐I signaling, we knocked out *Sting* expression by CRISPR/Cas9 technology to reduce type I IFN expression in tumor cells (Figure S6A,B, Supporting Information). When cocultured with DC cells, *Smad4*&*Sting* double KO partially diminished MHC‐I expression level and DC activation induced by *Smad4*KO coculture (**Figure** **5**A–C). On the other hand, *Smad4*KO cells‐induced DC activation was significantly attenuated in *Ifnar* ^−/−^ DCs (Figure 5D). In the tumor implantation model, *Smad4*KO‐induced tumor growth inhibition was attenuated when *Sting* was co‐deleted (Figure 5E). Consistently, T cell and DC infiltration and activation levels were significantly decreased in *Smad4&Sting*KO‐induced tumors comparing to those of *Smad4*KO tumors (Figure S6D, Supporting Information). Thus, tumor‐intrinsic STING‐mediated IFN‐I signaling is required for the *Smad4*KO‐induced antitumor response.

During an antitumor immune response, IFN‐I or DNA leaked from tumor cells also provoked IFN‐I signaling in immune cells, especially DCs, to form a positive feedback loop of such signaling.^[^ ^33^ ^]^ Indeed, DCs cocultured with *Smad4*KO PDAC cells, but not WT cells, exhibited upregulation of *Ifnα* and *Ifnβ* expression (Figure 5F), and anti‐IFNAR1 pretreatment abolished such effect (Figure 5G). Furthermore, when DCs were incubated with culture medium supernatant of tumor cells, the percentage of CD80^+^ cells also increased by supernatant from *Smad4*KO but not WT PDAC cells, nor *Smad4*&*Sting*KO cells (Figure S6C, Supporting Information), indicating the essential role of tumor‐intrinsic STING‐mediated signaling in DC activation. To test whether host STING signaling is required for *Smad4*KO‐induced tumor inhibition, we generated DC‐specific *Sting* knockout mice (*Itgax‐Cre*/*Sting^flox/flox^* mice) and challenged these mice with PDAC tumor cells. The result showed that STING deficiency in host DC cells had no impact on the tumor growth of *Smad4*KO cells (Figure 5H). Therefore, our findings showed that *Smad4*KO‐induced antitumor immunity was dependent on tumor cell‐intrinsic STING‐mediated IFN‐I signaling activation.

### 2.6 Restoration of *Smad4* Expression Partially Rescued *Smad4*KO‐Induced Tumor Inhibition

**Figure 6:**
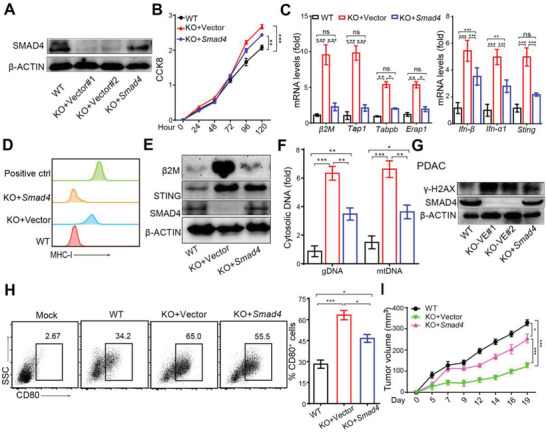
*Smad4* re‐expression in *Smad4*KO PDAC cells partially reversed tumor immunogenicity. A) Western blot analysis showing SMAD4 protein expression in PDAC cells. B) CCK8 assay showing cell proliferation of PDAC cells. Data are presented as mean ± SEM. Three independent experiments were performed. **, *p*< 0.01;***, *p* < 0.001; by two‐way ANOVA test. C) Real‐time qPCR analysis of mRNA levels of antigen presentation machinery molecules in PDAC cells. Three independent experiments were performed. Data are presented as mean ± SEM. ns, not significant, *, *p* < 0.05; **, *p* < 0.01;***, *p* < 0.001; by one‐way ANOVA test followed by Tukey's multiple comparison test. D) FCM analysis of MHC‐I expression levels on PDACs. E) Western blot analysis of *β*2M, STING and SMAD4 protein expression in PDAC cells. F) Cytoplasmic DNA and total DNA were isolated from tumor cells, and *Plog* (genomic DNA marker) and *Nd1*(mitochondrial DNA marker) gene levels were detected by real‐time PCR, and the ratio of cytoplasmic DNA is shown in fold change. Data are presented as mean ± SEM and three independent experiments were performed. *, *p*< 0.05; **, *p* < 0.01;***, *p* < 0.001; by one‐way ANOVA test followed by Tukey's multiple comparison tests. G) Western blot analysis of protein expression *γ*H2AX as DNA damage marker in PDAC cells. H) CD80^+^ cDCs were identified by FCM analysis after cocultured with PDAC tumor cells for 24 h, and the percentage of CD80^+^ cells in DCs is shown. At least three independent experiments were performed. Data are presented as mean ± SEM. *, *p* < 0.05; ***, *p* < 0.001; by one‐way ANOVA test followed by Tukey's multiple comparison test. I) Tumor growth of PDAC cells with indicated genotypes on B6 mice. Data are presented as mean ± SEM; *n* = 5 for each group. *, *p* < 0.05; ***, *p* < 0.001; by two‐way ANOVA test.

The results above showed that the deletion of *Smad4* in PDAC cells promoted tumor immunogenicity by increasing DNA damage‐induced IFN‐I signaling. To further verify that this is a tumor‐intrinsic *Smad4* function, we restored *Smad4* expression in *Smad4*KO cells (**Figure** **6**A). As expected, *Smad4*KO‐induced tumor cells’ hyper‐proliferation was inhibited by *Smad4* re‐expression (KO+*Smad4*) in vitro (Figure 6B). The expression levels of antigen presentation machinery molecules in *Smad4*KO cells were also reduced in *Smad4* re‐expressed cells (Figure 6C,D). However, the expression levels of IFN‐I or ISG genes in re‐expressed cells were partially reduced but still higher than WT cells (Figure 6C). In line with this, Western blot showed that increased *β*2M protein level in *Smad4*KO cells was reduced when *Smad4* was re‐expressed but STING expression level was still high (Figure 6E). Furthermore, cytoplasmic DNA levels and *γ*‐H2AX levels were decreased in *Smad*4KO cells when *Smad4* was re‐expressed, but were still higher than WT cells (Figure 6F,G). Enhanced CD80 expression on DCs induced by tumor cell coculture was significantly attenuated, but not completely reversed, in DCs cocultured with *Smad4* re‐expressed tumor cells (Figure 6H). Finally, *Smad4*KO‐mediated tumor growth inhibition was significantly, but not completely, reversed after *Smad4* re‐expression (Figure 6I). In summary, these data suggest that tumor‐intrinsic *Smad4* expression downregulated antigen presentation machinery gene expression and partially reduced DNA damage and IFN‐I pathway activity, together attenuated *Smad4*KO‐mediated tumor inhibition.

### 2.7 *SMAD4* Mutation and the Immune Microenvironment in Human PDACs

**Figure 7:**
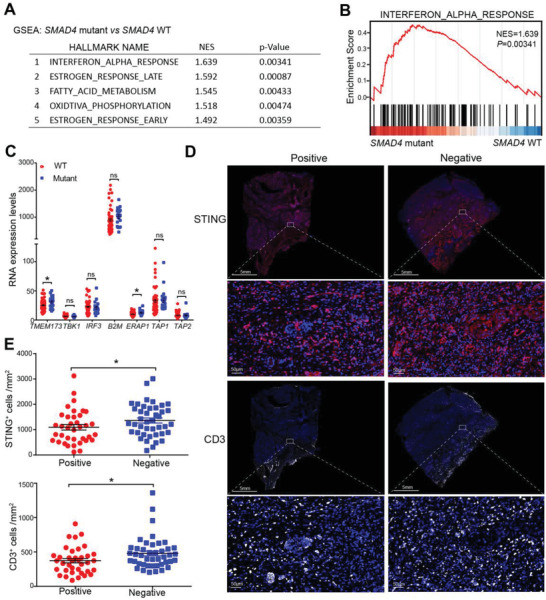
*SMAD4* mutation and the immune microenvironment in human PDACs. A,B) GSEA of gene expression between *SMAD4* mutant and *SMAD4* WT cases from TCGA (PAAD) database was performed. A) the significantly enriched hallmark pathways were listed; B) the IFN‐*α* response enrichment plots in patients was depicted. C) RNA expression levels of ISGs and antigen presentation machinery genes in TCGA database's pancreatic cancer patients selected by *KRAS* and *TP53* mutation. Data are presented as mean ± SEM; N*_SMAD4_*_WT_ = 64, N*_SMAD4_*_mutant_ = 20; ns，not significant, *, *p* < 0.05; by Mann–Whitney test. D,E) Multiplex‐IHC analysis of CD3 and STING expression on pancreatic cancer patients’ tumor tissues (N_SMAD4 negative_ = 43, *N*_SMAD4 positive_ = 38). Representative images were shown in (D), and the number of CD3^+^ and STING^+^ cells were counted and compared (E). Data are presented as mean ± SEM; *, *p* < 0.05; Mann–Whitney test.

Having established the biological function of *Smad4*‐loss in mouse pancreatic cancer cells, we next explored TCGA database cohort of pancreatic cancer patients for immune‐related gene expression. Among 139 cases of patients’ data retrieved from the database, 28 *SMAD4* mutant patients were identified. Although *SMAD4* mutation has been previously associated with radiotherapeutic resistance and poor survival in PDAC patients,^[^ ^31^, ^34^ ^]^ we did not observe a significant difference in the survival time between WT and *SMAD4* mutant groups (Figure S7A, Supporting Information). Gene set enrichment analysis (GSEA) identified that IFN‐*α* response signaling was enriched in *SMAD4* mutant group (**Figure** **7**A,B). Furthermore, among the ISG genes and antigen presenting machinery genes examined, *STING* and *ERAP1* gene expression levels were significantly higher in *SMAD4* mutant group than WT group among patients carrying *KRAS* and *TP53* muation (Figure 7C). CIBERSORT assay, which can estimate the cell proportions of major immune cell types from gene expression profiles,^[^ ^35^ ^]^ also revealed that resting and activated DC populations (Figure S7B, Supporting Information) were significantly increased in *SMAD4* mutant group. These results indicate that *SMAD4* mutant human pancreatic cancers showed stronger immunogenicity than that of *SMAD4* WT group.

We next evaluated the association of SMAD4 loss with the patients’ survival and immune infiltration using PDAC patient samples from the Sun Yat‐sen University Cancer Center (Guangzhou, China). Fifty SMAD4‐negative and 39 SMAD4‐positive pancreatic cancer patients were identified by IHC staining for SMAD4 expression. No significant difference in overall survival between patients with different SMAD4 expressions was observed (Figure S7E, Supporting Information). Immune infiltration (CD8^+^ cells, CD3^+^ cells, CD11c^+^ cells, Clec9a^+^ cells) and STING expression were also detected by multiplex IHC staining in pancreatic cancer patients’ tumor tissues (N_SMAD4 negative_ = 43, N_SMAD4 positive_ = 38). Compared with SMAD4‐positive group, the count of CD8^+^ and CLEC9A^+^ cells was modestly increased in the SMAD4‐negative group, and the number of CD3^+^ and STING^+^ cells in the SMAD4‐negative group was significantly higher than the SMAD4‐positive groups (Figure 7D,E; Figure S7C,D, Supporting Information). Together, loss of SMAD4 protein expression in human PDACs correlated with increased levels of STING expression and T cell infiltration in the tumor microenvironment by IHC staining.

## 3 Discussion

An effective anticancer immune response requires the release and presentation of tumor antigen from cancer cells to T cells. The presence of tumor‐specific antigens is associated with the proportion of tumor‐infiltrating lymphocytes (TILs) and sensitivity to antitumor immunotherapy. Poor immunogenicity enables tumor cells to evade the host's immunity and grow even in the presence of an intact immune system. Pancreatic cancer is reported to have a relatively low mutational load with a median somatic mutation and low MHC‐I levels.^[^ ^36^ ^]^ Yamamoto et al. recently found that autophagy in human PDAC cells degraded MHC‐I to promote tumor cells evasion, and inhibition of autophagy restored surface levels of MHC‐I and improved antigen presentation; leading to tumor growth inhibition in syngeneic host mice.^[^ ^28^ ^]^ Therefore, enhanced immunogenicity of PDAC is essential for improving antitumor immune response. Here, we found that the tumor growth of *Smad4* WT PDAC cells in vivo was not affected by the depletion of immune cells including CD4^+^, CD8^+^ T cells or NK cells, suggesting poor immunogenicity of the PDAC tumor cells. By contrast, *β*2M and MHC‐I levels were dramatically upregulated in PDAC cells after *Smad4* was knocked out and the tumor became sensitive to the host immune control of tumor growth. Interestingly, the increased MHC‐I levels in *Smad4*KO PDAC cells was not due to autophagy regulation as autophagy inhibition or stimulation did not change MHC‐I level much. Thus, multiple mechanisms exist for MHC‐I expression regulation in PDACs.

In addition to high‐frequency mutation in pancreatic cancer, *Smad4* is also inactivated or mutated at varying frequency in breast, colorectal and gastric cancer. In colorectal cancer, loss of *Smad4* facilitates liver metastasis by tumor accumulation of myeloid cells ^[^ ^37^ ^]^ and promotes cancer progression by increasing myeloid‐derived suppressor cells recruitment.^[^ ^38^ ^]^ Different from colorectal cancer, our findings suggest that *Smad4* loss enhanced PDAC tumor immunogenicity by eliciting DNA damage‐induced tumor‐intrinsic IFN‐I signaling and promoted immune cell infiltration. Thus, the function of *Smad4* in regulating tumor immunity may be tumor type‐ and context‐dependent. Consistent with our findings, Li et al. recently demonstrated that silencing *Smad4* in KPC pancreatic tumor cells accelerated T cell infiltration and suppressed implantation tumor growth.^[^ ^16a^ ^]^ Interestingly, when studying the epigenetic mechanism responsible for PDAC tumor progression, Hou et al. also observed striking tumor growth inhibition when *Smad4* expression was silenced in mouse pancreatic tumor cells with a *Kras^G12D^p53^−/−^* background.^[^ ^39^ ^]^

Our previous study reported that *Smad4* deletion increased DNA damage levels in human PDAC tumor cells.^[^ ^31^ ^]^ Mitra et al. reported that mice with keratinocyte‐specific *Smad4* deficiency exhibited increased DNA damage and increased UV‐induced skin cancer percentage by reducing DNA repair.^[^ ^15^ ^b]^ Consistently, head and neck epithelia‐specific *Smad4* KO mice exhibited increased genomic instability.^[^ ^15a^ ^]^ On one hand, accumulated DNA damage may result in genomic instability and promote carcinogenesis while on the other hand, DNA damage may result in the intracellular release of single‐ or double‐stranded DNA from mitochondria or nuclear genome,^[^ ^40^ ^]^ and cytosolic DNA can contribute to the immunogenicity of tumor cells.^[^ ^41^ ^]^ Cytoplasmic DNA is sensed by intracellular DNA sensors such as cGAS/STING or Toll‐like receptors (TLRs) which activates TBK1 and IRF3 to produce IFN‐Is and potentiates antitumor immunity by promoting DC cross‐presentation,^[^ ^42^ ^]^ and releases other proinflammatory cytokines.^[^ ^43^ ^]^ Given that *Smad4*KO‐mediated tumor inhibition is attenuated in *Ifnar* ^−/−^ mice, the essential role of the IFN‐I signaling pathway in PDAC tumor immunogenicity has been confirmed. STING is a critical adaptor for IFN‐I activation, and STING activation in the tumor can remodel the tumor vasculature, leading to increased sensitivity to immunotherapy, ^[^ ^44^ ^]^ and improve cross‐presentation of antigens by STING‐dependent adjuvants.^[^ ^45^ ^]^ In our animal tumor model, defective STING expression greatly promoted PDAC tumor growth in vivo. *Smad4*KO‐induced tumor inhibition was almost completely abrogated *by β2m* silencing, and partially by *Sting* KO. It indicates that increased *β*2M is critical for *Smad4*KO‐induced tumor growth inhibition and STING‐mediated IFN‐I signaling may act as an adjuvant for the antitumor immunity. In line with these findings, Benci et al. recently showed that in high‐antigenicity tumors, IFNs promoted CD8^+^ T cells and NK killing, but in poor‐antigenicity or MHC‐I‐low tumors, IFNs failed to potentiate CD8^+^ T cell killing.^[^ ^46^ ^]^

DCs can be subdivided into several subsets based on their dependence on specific transcription factors and diverse functional responses, phenotypic markers and tissue distribution, including conventional DCs (cDCs), plasmacytoid DCs (pDCs), Langerhans cells and monocyte‐derived DCs.^[^ ^47^ ^]^ cDCs, consisting of cDC1 and cDC2, efficiently initiate and cross‐presents antigens to CD8^+^ T or CD4^+^ T cells, respectively, making a significant contribution in antitumor immunity.^[^ ^47b^ ^]^ pDCs seem to be the primary IFN‐producing cells in innate antiviral immunity and the abundant production of type I interferon (IFN*α*/*β*) enhances cross‐priming of cDCs.^[^ ^48^ ^]^ Besides type I IFNs, type III IFNs produced by cDC1 predicts good clinical outcome.^[^ ^49^ ^]^ It is well established that CD103^+^ DC (i.e., cDC1) are major tumor antigen‐presenting cells in vivo for CD8^+^ T cells priming. In line with this, *Smad4*KO PDAC tumors have higher levels of infiltrated CD103^+^ DCs than WT tumors, and the growth disadvantage of *Smad4*KO cells was reversed in cDC1‐deficient *Batf3* ^−/−^ mice, suggesting a critical role of cDC1 in immune recognition and control of *Smad4*KO PDAC tumors. Consistently, cDC1 was found essential for T cell infiltration in PDAC tumors in another study characterizing the heterogeneity of the PDAC tumor microenvironment.^[^ ^50^ ^]^ Both tumor cell‐derived or host‐derived IFN‐I signaling contributes to antitumor immunity in vivo.^[^ ^51^ ^]^ Interestingly, DC‐specific *Sting* deficiency has no impact on WT or *Smad4*KO PDAC tumor growth, suggesting that tumor‐intrinsic STING/IFN‐I signaling, but not STING in DCs, is critical for *Smad4*KO‐mediated tumor inhibition.

In contrast with our findings from mouse PDAC tumor model, loss of *SMAD4* in human pancreatic cancer patients was frequently identified and was associated with poor survival.^[^ ^34^ ^]^ Such discrepancy may be due to the multiple functions of *SMAD4* in pancreatic cancer development, differentiation, immune regulation at different cancer stages. Early studies revealed that *SMAD4* deficiency did not affect normal pancreas development but accelerated PDAC development in combination with *KRAS* ^G12D^ mutation, and WT *SMAD4* in PDAC facilitated EMT and TGF‐*β*‐dependent growth.^[^ ^52^ ^]^ As the dual effect of TGF‐*β* on cancer development has been well illustrated,^[^ ^53^ ^]^ we reasoned that *S*MAD4’s function is also complex depending on multiple factors such as tumor stage, tissue context, and immune microenvironment. One recent study identified a four‐chemokine signature to predict PDAC immune infiltration and prognosis, indicating that T cell infiltration and antitumor immunity in human pancreatic cancers may require additional signals such as chemokines besides tumor immunogenicity.^[^ ^54^ ^]^ Interestingly, in line with different DC infiltration status found in *SMAD4* WT versus mutant PDAC cases in our CIBERSORT analysis, the authors also found that the chemokine signature was associated with STING expression and cDC1 marker expression. Nevertheless, human PDACs are highly resistant to anti‐PD1 or anti‐cytotoxic T lymphocyte‐associated antigen‐4 (CTLA4)‐based immunotherapy except a few microsatellite‐unstable cases, suggesting that SMAD4 loss alone could be insufficient to promote tumor response to checkpoint blockade. Recently *Smad4*‐deficient mouse PDACs were found highly sensitive to a combination immunotherapy regimen (regimen consisting of gemcitabine (G), abraxane (A), CD40 agonistic antibody (F), CTLA4‐blocking antibody (C), and PD‐1‐blocking antibody (P), referred to as GAFCP), mimicking a regimen currently undergoing clinical trial (NCT03214250).^[^ ^16a^ ^]^ Such findings also suggest that *SMAD4* status should be evaluated as a potential biomarker for tumor response to such therapy.

Nevertheless, there are a few questions that remain to be answered in this study. Firstly, whole exon sequencing suggests that the TMB level is not significantly different between WT and *Smad4*KO PDAC cells. We speculate that DNA damage level is not high enough to induce TMB accumulation and increased MHC‐I level in *Smad4*KO cells is largely due to IFN‐I pathway activation, but we cannot exclude that other pathways may contribute to MHC‐I upregulation. Secondly, re‐expression of *Smad4* failed to completely reverse *Smad4*KO‐induced tumor inhibition, and this may be attributed to persistent DNA damage and STING upregulation. Thirdly, pancreatic cancer stromal signaling is complex and is associated with dense structure and abnormal vasculature, leading to a highly hypoxic and nutrient‐poor environment of PDACs, which is disadvantageous for eliminating tumor cells.^[^ ^55^ ^]^ In RNA‐sequencing data of PDAC tumor cells, we noticed that a group of vasculature development‐related genes was enriched. Whether *Smad4*KO in PDAC cells also impacts tumor vasculature in the PDAC microenvironment needs to be further investigated.

Overall, our findings suggest that the poorly antigenic tumor cells of PDAC are resistant to immune control but deletion of *Smad4* increases MHC‐I expression on PDAC cells and promotes antitumor immune response. *Smad4* deficiency induces DNA damage and augments STING‐mediated IFN‐I signaling activation in tumor cells. The IFN‐I signaling, on one hand, increases MHC‐I expression and improves tumor cell antigenicity for enhanced immune recognition, while on the other hand reinforces the capacity of DC to cross‐priming T cells as an immune adjuvant. Our findings reveal a new link between *Smad4* deficiency and STING‐mediated IFN‐I signaling in PDACs, and suggest a potential role of SMAD4 status as a biomarker in developing future effective immunotherapy for PDACs.

## 4 Experimental Section

### Reagents

The following antibodies were used for Western blot analysis: anti‐SMAD4 (CST, 46535), anti‐STING (CST, 13647), anti‐RAD51 (CST, 8875), anti‐*β*2‐microglobulin (Abcam, ab75853), anti‐IRF3 (CST, 4302), anti‐phosphorylated‐IRF3 (CST, 29047), anti‐TBK1 (CST, 13504), anti‐phosphorylated‐TBK1 (CST, 5483), anti‐*γ*‐H2AX (CST, 9718), and anti‐*β*‐ACTIN (Sigma, A3854). Anti‐MHC‐I‐Eflour 450 (eBioscience, 48‐5999‐82) and anti‐MHC‐II‐FITC (eBioscience, 11‐5321‐82) were purchased from Thermo Fisher Scientific (USA). The neutralizing antibodies used for in vivo experiment were: Anti‐CD8 (BE0004‐1), anti‐CD4 (BE0003‐1), anti‐IFNAR1(BE0241), anti‐NK1.1(BE0036) and Rat lgG2a isotype control (BE0089) antibody, all purchased from BioXCell Inc. DMSO was obtained from Sigma‐Aldrich (St. Louis, MO), Murine IFN‐*β* was from R&D, Inc. (8234‐MB‐010), and murine TGF‐*β*1 was from Peptrotech Inc. (100‐21‐50).

### Cell Culture

All cell lines were routinely tested as mycoplasma‐negative. Murine pancreatic cancer (PDAC) cells were derived from primary pancreatic tumor tissues of a KPC mouse with mixed 129/C57BL/6 background, and the genotypes were determined by PCR using primers for mutant *Kras* activation (Kras‐F:5′‐GGGTAGGTGTTGGGATAGCTG‐3′, Kras‐R:5′‐TCCGAATTCAGTGACTACAGATGACAGAG‐3′) and *p53^R172H^* recombination (p53‐Re‐F: 5′‐AGC CTGCCTAGCTTCCTCAGG‐3′, p53‐Re‐R: 5′‐CTTGGAGACATAGCCACACTG‐3′).^[^ ^56^ ^]^ KPC‐1199 cells were derived from primary pancreatic tumor tissues of a KPC mouse with pure C57BL/6 background. The cells were cultured with DMEM (Invitrogen) supplemented with 10% FBS in 37 °C and 5% CO_2_ incubator.

For murine conventional dendritic cells (cDCs) isolation, bone marrow cells were isolated from 6 to 8 weeks old C57BL/6 mice and then cultured with 10% FBS RPMI 1640, containing 100 ng mL^−1^ FLT3L (Peprotech, 250‐31L). cDCs were cultured in a culture incubator at 37 °C with a 5% CO_2_ atmosphere for 7–9 days, and the culture medium was refreshed every 2 days.

### DC and T Cell Activation Assay

Equal numbers of tumor cells were cocultured with mature DCs for 24 h, RNAs from DCs were extracted to detect type I IFN expression. For DCs activation, DCs were stained with antibodies against CD11c (eBioscience, 17‐0114‐82), CD80 (Biolegend, 104706), CD86 (eBioscience, 48‐0862‐82), MHC‐I‐SIINFEKL (eBioscience, 17‐5743‐82) and analyzed by flow cytometry.

For T cells activation, PDAC‐OVA tumor cells were cocultured with DCs and B3Z T cells for 24 h. Supernatant level of IL‐2 was measured by an ELISA kit (eBioscience, 88‐7024‐88). OT‐I‐T cells were isolated from lymph nodes and spleens of OT‐I mice, then CD8 positive T cells were selected, and then T cells were cocultured with OVA‐expressing tumor cells for 24h, and the expression of IFN*γ* and Granzyme B were determined by flow cytometry.

### Animal Experiments

Female C57BL/6 mice (6‐8 weeks old) and Balb/c nude mice were purchased from Vital River Laboratory, Beijing, China. B‐NDG mice were purchased from Biocytogen Inc. *Ifnar* ^−/−^, *Batf3* ^−/−^ mice were purchased from Jackson Laboratory. *Sting^flox/flox^* mice were purchased from Shanghai Model Organism Inc. and crossed with *Itgax*‐*Cre* mice to obtain dendritic cell‐specific *Sting* knockout mice. All the mice were maintained in specific pathogen‐free conditions laboratory.

For tumor implantation experiments, 1 × 10^6^ PDAC cells were resuspend with 100 µL PBS and injected subcutaneously into the mouse. For orthotopic implantation models, 1 × 10^6^ PDAC cells were resuspended in 50 µL PBS and matrigel (1:1) mixture and orthotopically injected into mouse. For the antibody depletion experiment, CD4, CD8, IFNAR or NK1.1 depletion antibodies were intraperitoneally injected three times (100 µg per mouse). For KPC‐1199 tumor, 3 × 10^6^ cells were resuspended with 100 µL PBS:matrigel mixture (4:1), and injected subcutaneously into the mouse. All animal studies were performed in accordance with the National Institute of Health Guide for the Care and Use of Laboratory Animals with the approval of Sun Yat‐Sen University Cancer Center Institutional Animal Care and Use Committee.

### Pancreatic Cancer Patient Samples

Paraffin sections of tumor tissues from pancreatic cancer patients were obtained from the Sun Yat‐sen University Cancer Center. The study was performed in accordance with the Declaration of Helsinki and was approved by the Institutional Review Board of Sun Yat‐sen University Cancer Center and all patients were provided informed consent. The SMAD4 expression was detected by IHC staining, and the positive and negative status of SMAD4 expression in tumor tissues was evaluated by an experienced pathologist.

### CRISPR/Cas9‐Mediated Gene Knockout

*Smad4*, *Sting*‐deficient cells were constructed via the CRISPR/Cas9 technology.^[^ ^57^ ^]^ The sgRNA sequences were designed using the Optimized CRISPR Design (http://chopchop.cbu.uib.no/). The guide sequences used were listed as below: for *Smad4*: 5′‐GATCAGGCCACCTCCACAGA‐3′; 5′‐GGTGGCGTTAGACTCTGCCG‐3′, and for *Sting* 5′‐GTACCTTGGTAGACAATGAGG‐3′. The sgRNA was inserted into Lenti‐CRISPR v2 vector which contains the Streptococcus pyogenes Cas9 nuclease gene. The cells were selected with puromycin for 2 days after transiently transfected with Lenti‐CRISPR v2 vector, and the knockout effect was confirmed by Western blot.

### Western Blot Analysis

The cells were lysed in lysis buffer containing PhosSTOP phosphatase inhibitor Cocktail (Roche Diagnostics, Mannheim, Germany), and 1 mM DTT. Whole‐cell proteins were isolated by centrifuging for 12 000 × *g*, 5 min. Twenty micrograms of protein lysates was separated by SDS‐PAGE and then transferred to PVDF membranes. The membranes were then blocked in 5% BSA in PBST for 1 h at room temperature, followed by overnight incubation with the primary antibodies (1:1000 dilution); after three times of washing with PBST, the membranes were incubated with HRP‐conjugated secondary antibodies (1:2000) at room temperature for 1 h, and then the protein bands were visualized by chemiluminescence using an ECL detection kit (Thermo Scientific, 32106).

### Immunohistochemistry

For immunohistochemistry, tissue sections were deparaffinized in xylene (10 min for 3 times) and rehydrated by incubation in serial ethanol baths (100%, 95%, 90%, 80%, 70%, 5 min per bath). Epitope retrieval was performed through incubation in EDTA buffer (pH = 9.0) by boiling in a microwave for 10–20 min. Endogenous peroxidase activity was inhibited by treatment with 3% H_2_O_2_ for 10 min. The slides were then incubated overnight at 4 °C with anti‐CD8 (dilution: 1:200, CST, 98941), anti‐CD3 (dilution: 1:150, Abcam, 16669), anti‐CD4 (dilution: 1:100, ebioscience, 14‐8766‐82) and anti‐CD103 (dilution: 1:200, Abcam, ab224202) primary antibodies. After three times of washing in PBS, the slides were incubated for 30 min at room temperature with a secondary antibody (Dako, K5007), and the signal was subsequently detected by the chromogenic substrate (Dako, K5007). Slides were treated with hematoxylin for 3–5 min for nuclear staining, after which slides were treated with 1% hydrochloric acid ethanol (1% hydrochloric acid and 99% absolute ethyl alcohol) then dehydrated by incubation in serial ethanol baths (100%, 95%, 90%, 80%, 70%, 5 min per bath). The immunohistochemistry results of tissue slides were observed by microscope.

### Multiplex IHC Staining

PANO Multiplex IHC kit (Panovue) was used to examine specific cell markers including CD3 (Abcam, 16669), CD8 (ZSGB‐Bio, ZA0508), CD11c (CST, 45581), Clec9a (Abcam, ab223188), STING (CST, 13647). After the primary antibody was applied, the slides were incubated with secondary antibodies, followed by staining with fluorochrome (fluorochrome:tyramide signal amplification = 1:100). Next, the slides were subjected to microwave heat‐treated antigen retrieval in EDTA buffer (pH = 9.0) and then blocked with goat serum, followed by incubation with the next primary antibody. Nuclei were stained with DAPI after all of the antigens had been labeled. The stained slides were scanned using the Polaris Automatic Digital Slide Scanner (Akoya Biosciences, USA) which captured the fluorescence excitation spectrum at different wavelengths (480, 520, 620, 690, and 780 nm) within the proper exposure time. Multiple scans were combined to build a panoramic image. The images were analyzed and reconstructed images of sections by HALO image analysis software (lndica Labs, USA).

### Immunophenotyping Analysis of Tumor Microenvironment

WT and *Smad4*KO PDAC or KPC‐1199 tumor cells were subcutaneously injected into the flank of B6 mice. The tumors were allowed to grow for 20 days and were isolated and weighed. They were then dissociated by gentle MACS (Miltenyi Biotec) and filtered through 70 µm cell strainers to obtain single‐cell suspensions. The tumor signal cells were stained with antibodies against CD45 (BD, 563890), CD3 (Biolegend, 100236), CD4 (eBioscience, 63‐0042‐82), CD8 (eBioscience, 25‐0081‐82) CD69 (BioLegend, 104509), GzmB (ebioscience, 17‐8898‐82), IFN*γ* (eBioscience,17‐7311‐82) FOXP3 (BD,560401), ROR*γ*T (BD,562607), CD40 (eBioscience, 12‐0401‐82) and NK1.1 (eBiosicence, 12‐5941‐82) for T cell and NK cells analysis. For DC analysis, cells were stained with antibodies against CD45 (eBiosicence, 11‐0451‐82), CD11c (eBioscience, 45‐0114‐82), MHC‐II (invitrogen, MA1‐10403) and CD103 (BD, 562772). Fluorescence data were acquired on a BD LSR Fortessa Cytometer and analyzed using FlowJo, version 10. The number of cells counted by FCM analysis was divided by tumor weight to obtain the number of cells per gram of each tumor.

### Confocal Fluorescence Microscopy

The cells were fixed with 4% paraformaldehyde for 15 min, after which the cells membrane were permeabilized by 0.2% Triton X‐100 for 10 min, followed by 10% BSA‐PBS blocking for 40 min, and incubated with anti‐*γ*‐H2AX (1:200, CST, 9718) overnight at 4°C. The cells were washed with PBS, then incubated with FITC‐conjugated secondary antibodies (CST, 4412) for 40 min at room temperature. The nuclei were stained with DAPI for 10 min at room temperature. The images were obtained by a confocal microscope (Zeiss Axiovert, LSM880).

### Detection of Genomic and Mitochondrial DNA in Cytosolic Extracts

Cytosolic DNA was extracted and the procedure for cytoplasmic DNA detection in the cytoplasm was performed as previously described,^[^ ^40^ ^]^ and quantified genomic DNA and mitochondrial DNA via quantitative PCR (qPCR) using the primer specific for genomic DNA (*Polg1*) and mitochondrial DNA (*Nd1*). The primer sequence of *polg1* was as follows: forward primer, 5′‐GATGAATGGGCCTACCTTGA‐3′, and reverse primer, 5′‐TGGGGTCCTGTTTCTACAGC‐3′. For mitochondrial DNA (*Nd1*): forward primer, 5′‐CAAACACTTATTACAACCCAAGAACA‐3′, and reverse primer, 5′‐TCATATTATGGCTATGGGTCAGG‐3′.

### *Smad4* Expression by Lentiviral Vector

The DNA fragments from the CDS sequence of murine *Smad4* (NM_008540.3) were obtained by PCR, inserted into the vector pCDH‐coGFP‐T2A‐puro, then lentiviruses expressing murine *Smad4* were generated (Forward primer, 5′‐AATTCATGGACAATATGTCTATAACAAATACACCAACAAGTAAC‐3′, and reverse primer, 5′‐TGCTCTAGATCAGTCTAAAGGCTGTGGGTCCGCAA‐3′). PDAC‐*Smad4*KO cells were infected with this virus, and the positive cells were selected by puromycin and flow cytometry (BD Biosciences, San Jose, CA). The PDAC‐Vector cells infected with empty vector were used as a control.

### RNA Isolation and Real‐Time Quantitative PCR

Total RNA was extracted using Trizol (Life Technologies, Carlsbad, CA), and cDNA was obtained using a one‐step cDNA synthesis kit TransScript One‐Step gDNA Removal and cDNA Synthesis SuperMix (TransGen Biotech, Beijing, P. R. China). Then, real‐time quantitative PCR (qRT‐PCR) was performed to quantitative relative mRNA levels using the SYBR Select Master Mix (Life Technologies) on Mastercycler ep realplex (Eppendorf, Hamburg, Germany). The sequences of primers used were listed in Table S3 (Supporting Information).

### RNA Sequencing and Whole Exon Sequencing

WT and *Smad4*‐deficient PDAC cells were collected and total RNA was extracted using Trizol (Life Technologies, Carlsbad, CA) following the manufacturer's protocol, Total RNA was submitted to Fulgent Technologies Inc (Fujian, China) for RNA‐sequencing using HiSeq X ten/NovaSeq (Illumina). Raw reads were screened using Trimmomatic to obtain clean reads and were mapped to corresponding mouse genome informatics database (The Jackson Laboratory) by using HISAT.

For whole exon sequencing, the cells were collected and DNA was isolated for sequencing using Illumina PE150 by Jiangxi Haplox Clinical Laboratory Center, Ltd (Jiangxi, China). Clean reads were obtained by using fastb and were compared with mouse genome informatics database (The Jackson Laboratory) through sentieon BWA. SNP/INDEL was examined using the GATK software and somatic SNV/INDEL was analyzed using MuTect2. TMB was calculated after synonymous and nonsense mutation was excluded.

### TCGA Cohort Analyses

The cohort of pancreatic cancer was of epithelial origin including adenocarcinoma and squamous cell carcinoma. *SMAD4* mutant case was sorted using the exome sequencing data. After *SMAD4* stop‐gain and loss of function mutation cases were selected, RNA‐seq data of those were subjected to GSEA (https://www.gsea‐msigdb.org/gsea/index.jsp) and CIBERSORT assay analysis. *SMAD4* wild‐type cases were used as control.

## Conflict of Interest

The authors declare no conflict of interest.

## Author Contribution

W.X. and W.H. contributed equally to this work. W.X. refined the idea, performed research, analyzed data and wrote the manuscript; W.X. and W.H. performed experiments and analyzed data with technical assistance from T.W., F.X., Z.W., X.W., H.G., H.Z., Y.L., K.X., M.L., H.Z., J.Li.; J.H.L. and J.X. provided key experiment reagents; S.H., N.N., Q.Z., Z.‐X.L., J.‐X.B. performed bioinformatics analysis; P.H. provided reagents and manuscript revising; J.L. and L.X. contributed to manuscript revising and project supervision; X.X. designed and supervised the project, obtained funding and revised the manuscript.

## Supporting information

Supporting Information

## Data Availability

The data that support the findings of this study are available from the corresponding author upon reasonable request.
