# Supplementary material for: Smad4 Deficiency Promotes Pancreatic Cancer Immunogenicity by Activating the Cancer‐Autonomous DNA‐Sensing Signaling Axis
Source: Adv Sci (Weinh). 2022 Jan 22;9(7):2103029. doi: 10.1002/advs.202103029 (PMC8895117; doi:10.1002/advs.202103029)
Supplement: Supplementary file 1 — Supporting Information [file ADVS-9-2103029-s001.pdf]

ADVANCED SCIENCE
Open Access

Supporting Information

for *Adv. Sci.,* DOI: 10.1002/advs.202103029

Smad4 Deficiency Promotes Pancreatic Cancer Immunogenicity by Activating the Cancer-Autonomous DNA-Sensing Signaling Axis

Wenjing Xiong, Wenzhuo He, Tiantian Wang, Shuai He, Feifei Xu, Zining Wang, Xiaojuan Wang, Hui Guo, Jianhua Ling, Huanling Zhang, Yongxiang Liu, Kaili Xing, Mengyun Li, Hongxia Zhang, Jiahui Li, Ningning Niu, Jing Xue, Qiuyao Zhan, Ze-Xian Liu, Jin-Xin Bei, Peng Huang, Jinyun Liu, Liangping Xia,* and Xiaojun Xia**

Supplement Figures

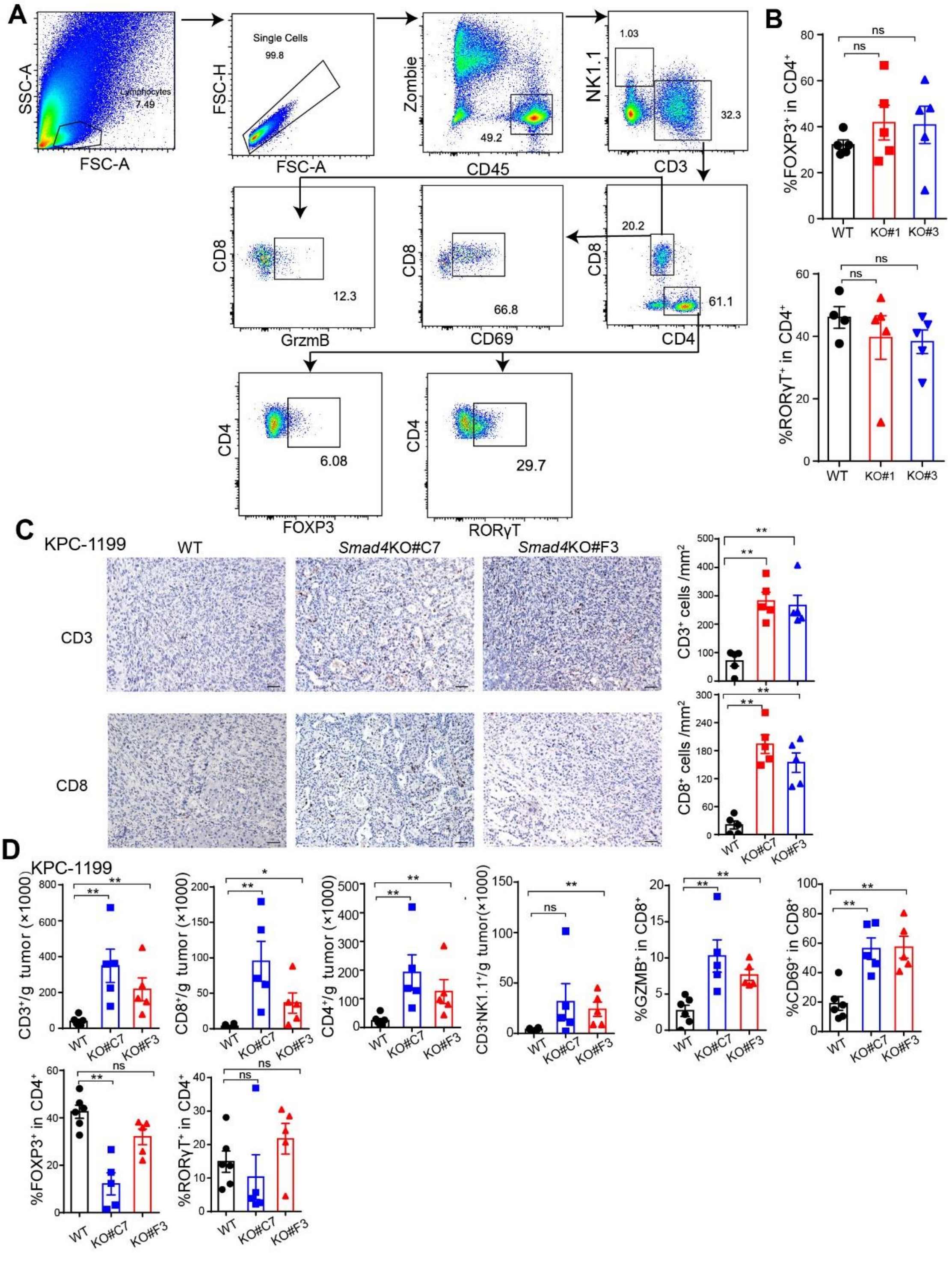

Supplement Figure 1. *Smad4* deletion in PDAC induced immune infiltration in tumor tissues.

(A) Gating strategies for FCM analysis of single cells from tumor tissues used in this study.

(B) Single cell suspension isolated from WT or *Smad4*KO PDAC tumors was stained

and gated with CD4$^+$FOXP3$^+$, CD4$^+$RORγT$^+$ cells to detect Treg and Th17 cells and the percentage were calculated. Data are presented as mean ± SEM, n=5, *, $p < 0.05$; **, $p < 0.01$; by Mann-Whitney test.

(C) Left, representative IHC staining images of the WT and *Smad4*KO (two clones: KO#C7 and KO#F3) KPC-1199 tumor tissues stained with anti-CD3 or anti-CD8, Scale bar=20 μm. Right, the number of CD3$^+$ and CD8$^+$ cells per mm^2 was counted. *, $p < 0.05$; **, $p < 0.01$; by Mann-Whitney test.

(D) Single cell suspensions isolated from WT or *Smad4*KO KPC-1199 tumors were stained and FCM analysis was performed for immunotyping. Data are presented as mean ± SEM, n=5 or 6, *, $p < 0.05$; **, $p < 0.01$; by Mann-Whitney test.

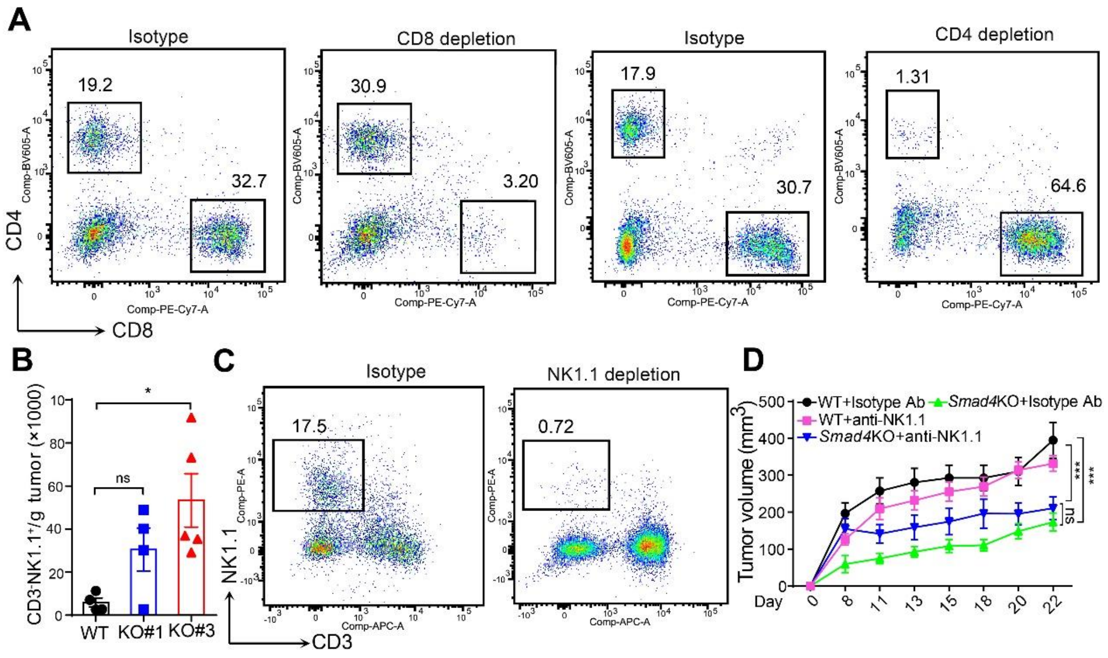

Supplement Figure 2. T cells and NK cells deletion in PDAC mouse tumor model.

(A) The depletion of CD4$^+$ and CD8$^+$ cells by neutralizing antibodies were confirmed by FCM analysis using tumor tissues.

(B) C57BL/6 mice were injected with WT or *Smad4*KO PDAC cells subcutaneously, and tumor tissues were isolated after 22 days and prepared into single cell suspension for FCM analysis. The number of CD3$^-$NK1.1$^+$ cells per gram tumor was counted. n=4 or 5 per group, *, $p < 0.05$; by Mann-Whitney test.

(C and D) NK cells in host mice were depleted by intraperitoneal injection of anti-NK1.1 neutralizing antibody prior to tumor cell inoculation. NK deletion was examined by FCM analysis (C), and tumor growth was monitored (D). Data are presented as mean ± SEM; n=5, ***, $p < 0.001$; by two-way ANOVA test.

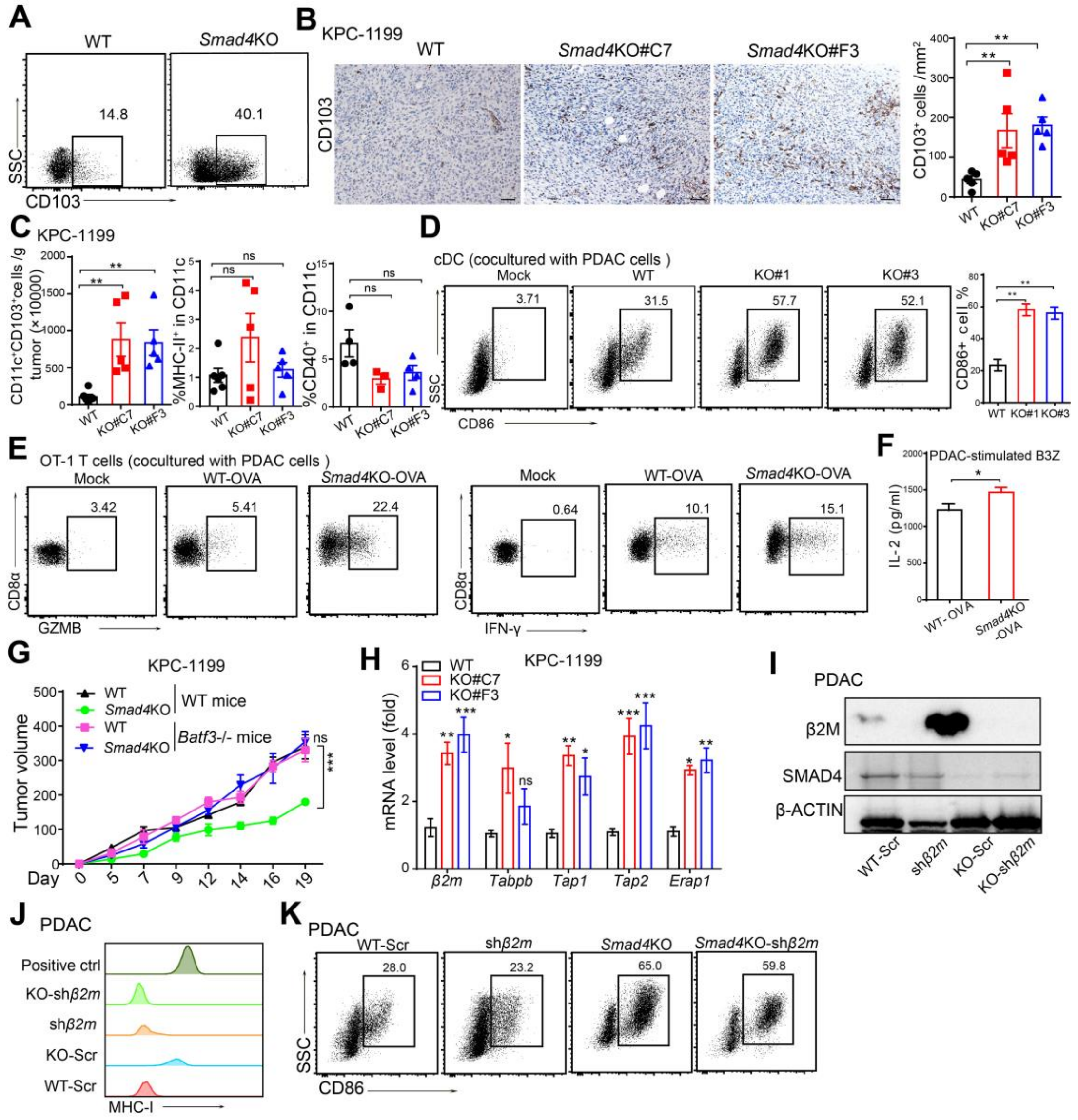

Supplement Figure 3. *Smad4* deficiency in PDAC cells enhanced tumor immunogenicity.

(A) Intratumor $CD103^+$ cell frequencies among $CD11c^+$ cells in WT or *Smad4*KO PDAC tumors by FCM analysis.

(B) Left, representative anti-CD103 IHC staining images of the WT and *Smad4*KO KPC-1199 tumor tissues. Right, bar graph showing the count of $CD103^+$ cells per

mm^2, Scale bars=20 μm. Data are presented as mean ± SEM. **, $p < 0.01$; by Mann-Whitney test.

(C) $CD11c^+ CD103^+$ cells and the percentage of MHC-II^+ and $CD40^+$ DCs in WT or *Smad4*KO KPC-1199 tumors were identified by FCM analysis. Data are presented as mean ± SEM; ns, not significant; **, $p < 0.01$; by Mann-Whitney test.

(D) cDCs were cocultured with PDAC cells (WT-control or *Smad4*KO) for 24 h, and the percentage of $CD86^+$ DCs was identified by FCM analysis. Data are presented as mean ± SEM. **, $p < 0.01$; by Mann-Whitney test.

(E) WT-OVA and *Smad4*KO-OVA PDAC cells were cocultured with OT-I T cells, and the frequencies of IFN-γ^+ and Granzyme B^+ (GZMB) cells in $CD8^+$ T cells was identified by FCM analysis.

(F) WT-OVA and *Smad4*KO-OVA PDAC cells were cocultured with DCs and B3Z T cells, and IL-2 levels in culture supernatant was detected by ELISA. Three independent experiments were performed. Data are presented as mean ± SEM. *, $p < 0.05$; by Student's t test.

(G) KPC-1199 cells were subcutaneously injected into WT or $Batf3^{-/-}$ mice, and then tumor growth was monitored at the indicated time points. Data are presented as mean ± SEM; n = 5 per group. ns, not significant; ***, $p < 0.001$; by two-way ANOVA test.

(H) Real-time PCR analysis of expression levels of antigen presentation machinery genes in KPC-1199 cells. Data are presented as mean ± SEM; three independent experiments were performed. ns, not significant; *, $p < 0.05$; **, $p < 0.01$; ***, $p < 0.001$; by Mann-Whitney test.

(I) Western blot analysis of β2M protein expression levels in PDAC cells.

(J) FCM analysis detecting the surface MHC-I expression in PDAC cells.

(K) cDCs were cocultured with PDAC cells (WT-control, sh*β2m*, *Smad4*KO and Smad4KO&sh*β2m*) for 24 h, and the percentage of $CD86^+$ DCs was identified by FCM analysis.

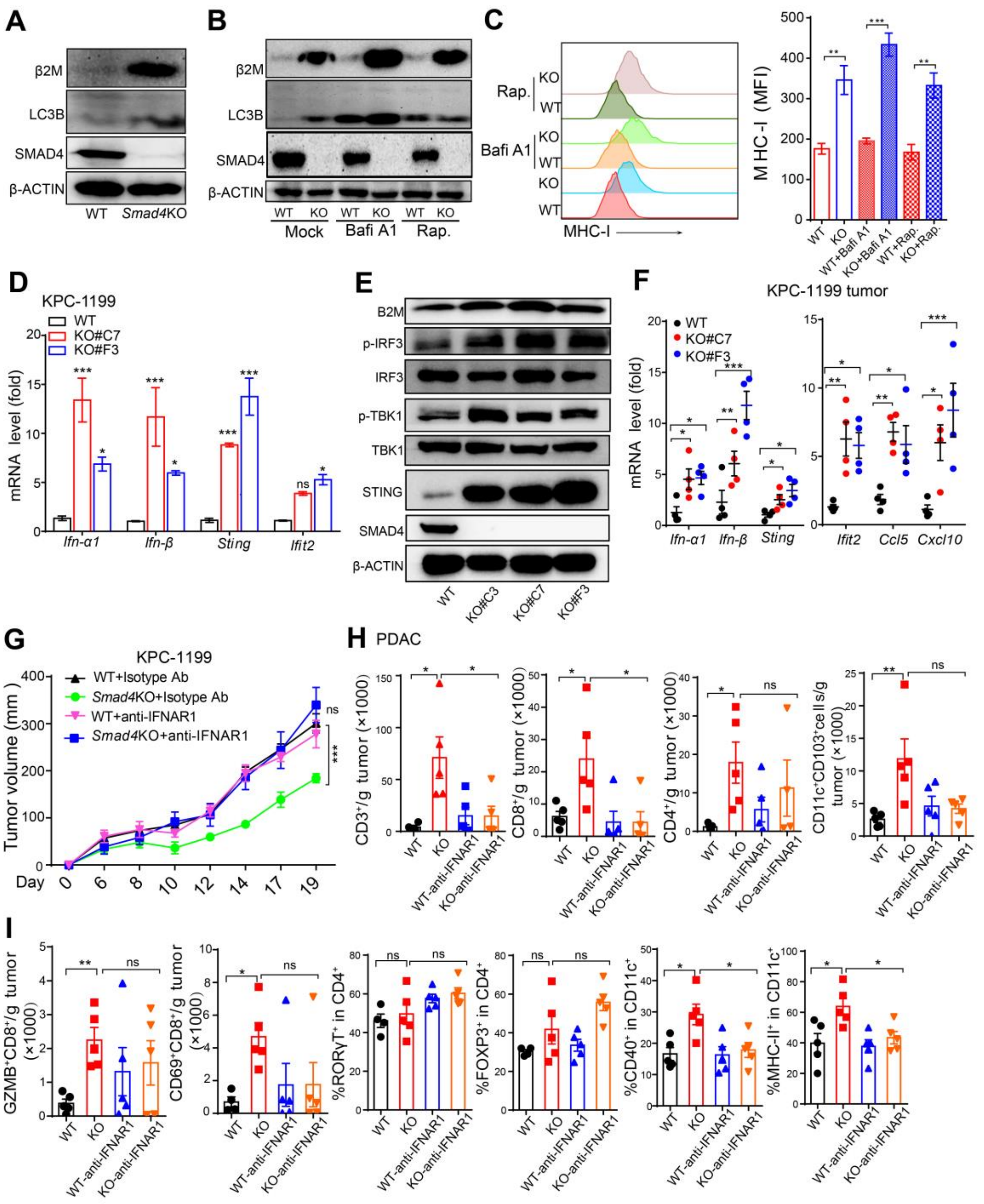

Supplement Figure 4. ***Smad4*** **deletion enhanced PDAC tumor immunogenicity by activating IFN-I signaling.**

(A) Western blot analysis showing LC3B and β2M protein levels in WT and *Smad4*KO PDAC cells.

(B) Western blot showing LC3B and β2M protein expression levels in PDAC cells with or without treatment of Rapamycin (Rap.) or Bafilomycin A1 (Bafi A1) for 18 h.

(C) FCM analysis showing surface MHC-I expression levels (indicated by Mean Fluoresence Intensity (MFI)) in PDAC cells with or without treatment of Rapamycin

or Bafilomycin A1 for 18 h. Right, bar graph showing the quantified results of MHC-I expression levels. Data are presented as mean ± SEM; **, $p< 0.01$; ***, $p < 0.001$; by Mann-Whitney test.

(D) Real-time PCR analysis of mRNA levels of ISGs in KPC-1199 cells. Data are presented as mean ± SEM; ns, not significant; *, $p < 0.05$; ***, $p < 0.001$; by Mann-Whitney test.

(E) Western blot analysis of protein expression levels in WT and *Smad4*KO KPC-1199 cells.

(F) RNA from WT and *Smad4*KO KPC-1199 tumor tissues were isolated and real-time PCR was performed to detect expression of ISGs as indicated. Data are presented as mean ± SEM; n = 4 tumors each group. *, $p < 0.05$; **, $p< 0.01$; ***, $p < 0.001$; by Mann-Whitney test.

(G) KPC-1199 tumor growth on C57BL/6 mice with IFNAR1 blockade by intraperitoneal injection of anti-IFNAR1 antibody. Data are presented as mean ± SEM; n = 5 for each group. ns, not significant; ***, $p < 0.001$; by two-way ANOVA test.

(H-I) T cell and DC infiltration and activation levels in anti-IFNAR1-treated PDAC tumors were examined by FCM analysis. Data are presented as mean ± SEM; ns, no significant; *, $p < 0.05$; **, $p< 0.01$; by Mann-Whitney test.

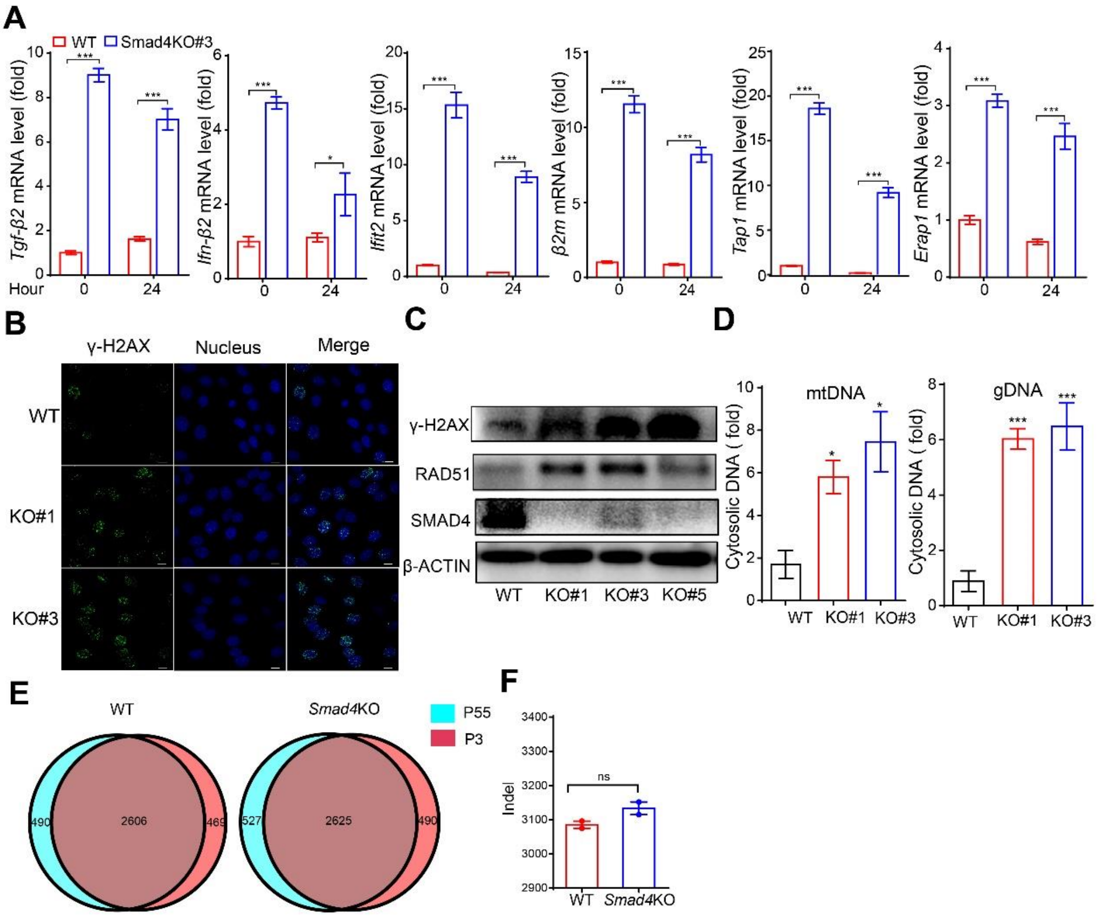

Supplement Figure 5. *Smad4* deletion enhanced PDAC tumor immunogenicity by inducing DNA damage.

(A) Real-time PCR analysis of mRNA levels of indicated genes in TGF-β1-treated cells. Data are presented as mean ± SEM. *, $p < 0.05$; ***, $p < 0.001$; by Mann-Whitney test.

(B) Representative immunofluorescent staining images of γ-H2AX in PDAC cells detected by confocal microscopy. Scale bars=10 μm.

(C) Western blot showing protein expression of γ-H2AX and RAD51 in PDAC cells.

(D) Cytoplasmic DNA and total DNA were isolated from WT or *Smad4*KO PDAC cells, and *Plog* (genomic DNA marker) and *Nd1* (mitochondrial DNA marker) gene levels were detected by real-time PCR, and the ratio of cytoplasmic DNA is shown in fold change. Data are presented as mean ± SEM; three independent experiments were performed. *, $p < 0.05$; ***, $p < 0.001$; by Mann-Whitney test.

(E) The numbers of indels (insertion and deletion mutation) in WT and *Smad4*KO

PDAC cells with different passages (P3 and P55).

(F) The statistical analysis of the indels between WT and *Smad4*KO PDAC cells. ns, no significant; by Mann-Whitney test.

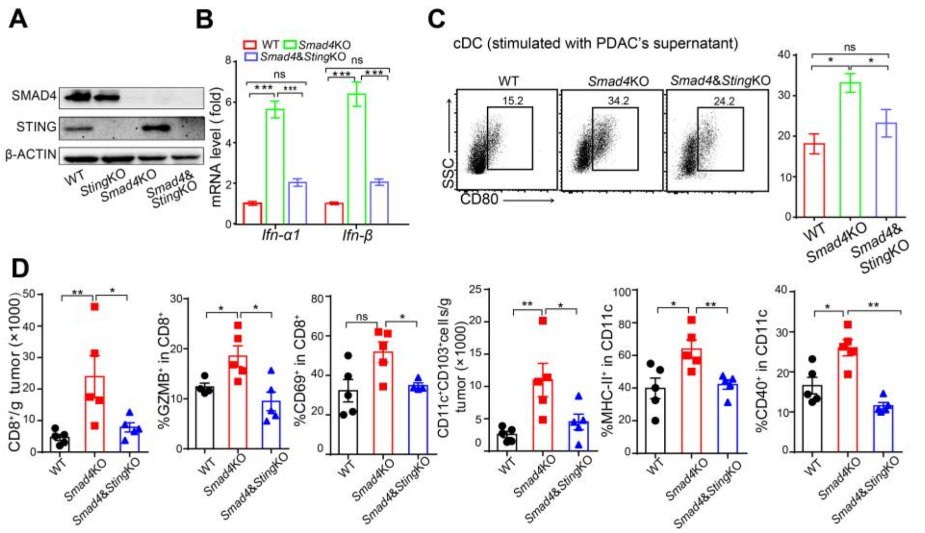

Supplement Figure 6. The role of STING in *Smad4* deletion-induced PDAC tumor immunogenicity.

(A) Western blot analysis showing SMAD4 and STING protein expression levels in PDAC cells.

(B) Real-time qPCR examining mRNA levels of IFN-I genes in PDAC cells. Three independent experiments were performed. Data are presented as mean ± SEM. ns, not significant; ***, $p<0.001$; by Mann-Whitney test.

(C) Left, murine DCs were cocultured with culture medium supernatant from tumor cells (WT, *Smad4*KO, and *Smad4&Sting*KO) for 24 h, and the frequency of $CD80^+$ DCs was shown as indicated. Right, bar graph showing quantified frequencies of $CD80^+$ DCs in different groups. Three independent experiments were performed. Data are presented as mean ± SEM. ns, not significant; *, $p < 0.05$; by Mann-Whitney test.

(D) T cell and DC infiltration and activation levels in PDAC tumors were examined by FCM analysis. Data are presented as mean ± SEM; ns, not significant; *, $p < 0.05$; **, $p < 0.01$; by Mann-Whitney test.

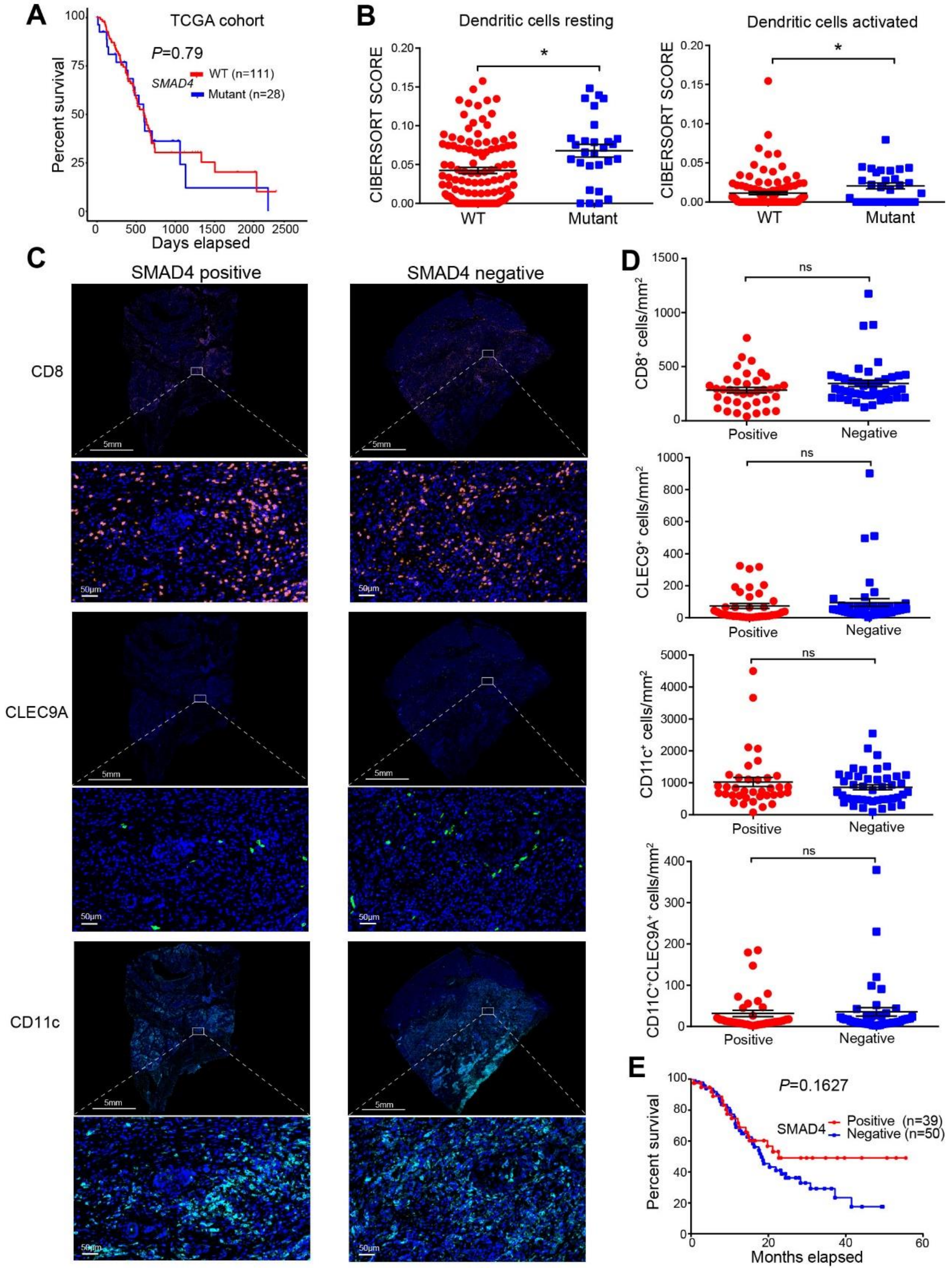

Supplement Figure 7. SMAD4 expression and immune microenvironment in human PDAC tumor tissues.

(A) Survival curve of pancreatic cancer patients from TCGA cohort, *p*=0.79, by Gehan-Breslow-Wilcoxon test. $N_{\textit{SMAD4}\ \mathrm{WT}}$=111, $N_{\textit{SMAD4}\ \mathrm{mutant}}$=28.

(B) CIBERSORT analysis comparing tumor-infiltrated dendritic cell population levels in *SMAD4*-mutant and *SMAD4*-WT pancreatic cancer patients from TCGA database.

Data are presented as mean ± SEM; $N_{\textit{SMAD4}\ \mathrm{WT}}$=111, $N_{\textit{SMAD4}\ \mathrm{mutant}}$=28; *, $p < 0.05$; by Mann-Whitney test.

(C and D) C, the representative images of CD8, CLEC9A, and CD11C staining on tumor tissue slides from pancreatic cancer patients using multiplex-IHC staining, and the quantified results comparing positive cell frequencies in SMAD4-positive and SMAD4-negative samples were shown in D. $N_{\mathrm{SMAD4\ negative}}$=43, $N_{\mathrm{SMAD4\ positive}}$=38, data are presented as mean ± SEM; ns, not significant, by Mann-Whitney test.

(E) Survival of SMAD4-positive and SMAD4-negative pancreatic cancer patients, ($N_{\mathrm{SMAD4\ negative}}$=50, $N_{\mathrm{SMAD4\ positive}}$=39), p=0.1627, by Log-rank (Mantel-Cox) test.

Supplement Table 1. TMB levels of WT and *Smad4*KO PDAC cells

**Sample**	**TMB (mutations per Mb)**
WT-P5	118.0644
WT-P55	118.0847
*Smad4*KO-P3	118.7125
*Smad4*KO-P55	119.0567

Supplement Table 2. SNV levels of WT and *Smad4*KO PDAC cells

Type	WT-P3	WT-P55	*Smad4*KO-P3	*Smad4*KO-P55
CDS	198	201	198	208
Frame shift deletion	39	4<br>0	38	40
Frame shift insertion	23	26	24	24
Non-frame shift deletion	73	7<br>1	68	75
Non-frame shift insertion	49	52	54	55
Stop-gain	2	1	2	1
Stop-loss	0	0	0	0
unknown	12	1<br>1	12	13
intronic	2,331	2,346	2,376	2,400
UTR3	170	176	161	168
UTR5	48	4<br>6	53	47
splicing	14	13	12	12
ncRNA exonic	63	6<br>4	53	58
ncRNA intronic	55	54	54	53
ncRNA UTR3	0	0	0	0
ncRNA UTR5	0	0	0	0
ncRNA splicing	0	0	0	0
upstream	21	25	24	20
downstream	37	35	37	39
intergenic	138	136	147	147
Total	3,075	3,096	3,115	3,152

Supplement Table 3. Sequences of primers used for qPCR

Gene	forward primer (5'-3')	reverse primer (5'-3')
β2m	TTCTGGTGCTTGTCTCACTGA	CAGTATGTTCGGCTTCCCATTC
Tap1	GGACTTGCCTTGTTCCGAGAG	GCTGCCACATAACTGATAGCGA
Tapbp	GGCCTGTCTAAGAAACCTGCC	CCACCTTGAAGTATAGCTTTGGG
Earp1	TAATGGAGACTCATTCCCTTGGA	AAAGTCAGAGTGCTGAGGTTTG
Ifnα1	GCCTTGACACTCCTGGTACAAATGAG	CAGCACATTGGCAGAGGAAGACAG
Ifn-β	CAGCTCCAAGAAAGGACGAAC	GGCAGTGTAACTCTTCTGCAT
Isg15	GGTGTCCGTGACTAACTCCAT	TGGAAAGGGTAAGACCGTCCT
Ifit2	AGTACAACGAGTAAGGAGTCACT	AGGCCAGTATGTTGCACATGG
Sting	GGTCACCGCTCCAAATATGTAG	CAGTAGTCCAAGTTCGTGCGA
Ccl5	GCTGCTTTGCCTACCTCTCC	TCGAGTGACAAACACGACTGC
Cxcl10	CCAAGTGCTGCCGTCATTTTC	GGCTCGCAGGGATGATTTCAA
β-Actin	AGAGGGAAATCGTGCGTGAC	CAATAGTGATGACCTGGCCGT
Tgf-β2	TCGACATGGATCAGTTTATGCG	CCCTGGTACTGTTGTAGATGGA
